# Structural Model for the Interaction of a Designed Ankyrin Repeat Protein with the Human Epidermal Growth Factor Receptor 2

**DOI:** 10.1371/journal.pone.0059163

**Published:** 2013-03-19

**Authors:** V. Chandana Epa, Olan Dolezal, Larissa Doughty, Xiaowen Xiao, Christian Jost, Andreas Plückthun, Timothy E. Adams

**Affiliations:** 1 Commonwealth Scientific & Industrial Research Organization Materials Science & Engineering, Parkville, Victoria, Australia; 2 Biochemisches Institut der Universität Zürich, Zürich, Switzerland; University of Edinburgh, United Kingdom

## Abstract

Designed Ankyrin Repeat Proteins are a class of novel binding proteins that can be selected and evolved to bind to targets with high affinity and specificity. We are interested in the DARPin H10-2-G3, which has been evolved to bind with very high affinity to the human epidermal growth factor receptor 2 (HER2). HER2 is found to be over-expressed in 30% of breast cancers, and is the target for the FDA-approved therapeutic monoclonal antibodies trastuzumab and pertuzumab and small molecule tyrosine kinase inhibitors. Here, we use computational macromolecular docking, coupled with several interface metrics such as shape complementarity, interaction energy, and electrostatic complementarity, to model the structure of the complex between the DARPin H10-2-G3 and HER2. We analyzed the interface between the two proteins and then validated the structural model by showing that selected HER2 point mutations at the putative interface with H10-2-G3 reduce the affinity of binding up to 100-fold without affecting the binding of trastuzumab. Comparisons made with a subsequently solved X-ray crystal structure of the complex yielded a backbone atom root mean square deviation of 0.84–1.14 Ångstroms. The study presented here demonstrates the capability of the computational techniques of structural bioinformatics in generating useful structural models of protein-protein interactions.

## Introduction

The human epidermal growth factor receptor 2 (HER2 or ErbB2) is over-expressed in several cancers, in particular in about 30% of breast tumors [1] and is indicative of a poor prognosis for these patients. Members of the HER/EGFR family are cell-surface receptors that have an intracellular tyrosine kinase domain and an ectodomain consisting of four distinct domains including the ligand-binding regions (domains 1 and 3) and two cysteine-rich domains (domains 2 and 4). HER2 plays a fundamental role in normal growth and development, eliciting a complex program of intracellular signaling and cellular responses, initiated by heterodimerization with other members of the HER family, in particular HER3 and HER4 [2], that become dimerization competent through ligand binding. Over-expression of HER2, usually the result of gene amplification, allows the constitutive dimerization of the receptor with HER3 devoid of ligand [3] and all liganded members of the EGFR family, and is thus a major factor in the development and maintenance of malignancy. Hence, HER2 is an important target for cancer therapeutic and diagnostic development. Of the HER2 binding monoclonal antibodies that are used in the clinic, trastuzumab (Herceptin™) binds to domain 4 [4] while pertuzumab (Perjeta™) binds to domain 2 [5].

Designed ankyrin repeat proteins (DARPins) [6], [7], [8] are a novel class of small, highly stable binding proteins that can be selected by ribosome display to bind target proteins with high affinity and can be expressed in bacteria in high yields. Because of their small size, DARPins targeting cell-surface proteins are expected to have much better tissue penetration and higher clearance than antibodies recognizing the same protein target when administered *in vivo*. The DARPin H10-2-G3 (hereafter referred to as ‘G3’) has been selected to bind HER2 with picomolar affinity [9]. This DARPin has been found to be as reliable and even more specific when compared to an FDA-approved anti-HER2 antibody used for testing the status of HER2 in paraffin-embedded breast cancer tissue [10]. G3 consists of only two ankyrin repeat motifs flanked by N-terminal and C-terminal capping regions. Each of the 33-residue ankyrin repeat motifs contain two antiparallel α-helices. G3 has been randomized at six positions in each of the repeats and contains four further mutations in the framework region. G3, while binding to domain 4 of HER2 [10], does not compete with trastuzumab in binding to HER2 (C. Jost and A. Plückthun, unpublished data). Tumor targeting experiments with mice bearing HER2-overexpressing human breast cancer xenografts have shown high tumor accumulation correlating with the affinity of the DARPins to HER2 [11].

Knowledge of the three-dimensional structure of the G3-HER2 complex would be very valuable in understanding the structural basis of the interaction between the DARPin and HER2 and would facilitate protein engineering approaches for anti-HER2 DARPins that contain G3. In the current paper, we use macromolecular computational docking methodology in combination with a number of different energetic and structural metrics to construct a 3-dimensional atomic structural model of the complex between G3 and HER2. We then selected putative interacting amino acid residues on HER2 to mutagenize. By analyzing the impact of these mutations on the interaction of G3, we provide evidence that validates the structural model of the G3-HER2 complex. Structural comparison with a subsequently solved X-ray crystal structure (PDB id: 4HRN) of the complex provides a quantitative measure of the accuracy of the computational model.

## Results and Discussion

### Modeling the Structure of the G3-HER2 Complex

The construction of the three-dimensional structure of the complex between HER2 and G3 commenced with the X-ray crystal structure of HER2 in complex with trastuzumab [4] and the X-ray crystal structure of the HER2-specific DARPin G3 [9]. G3 is one of the KEYL sequence family DARPins evolved by affinity maturation and exhibits affinity of 90 pM to HER2. As Cho et al. [4] describe in their crystal structure of the complex between trastuzumab-Fab and the HER2 ectodomain, trastuzumab binds to the domain 4 of HER2. While G3 also binds to domain 4 of HER2, it does not compete with trastuzumab in binding to the receptor (C. Jost and A. Plückthun, unpublished). For this reason we used only domain 4 (residues #508–607) in macromolecular docking to construct the structural model of the G3-HER2 complex. This also has the effect of making the computational task more tractable and facilitates the generation of more accurate results through the use of a finer grid mesh for the docking than otherwise would have been possible. Chain A (residues #12–135) of the G3 structure was used in the modeling. Prior to the macromolecular docking, the structure of the loop containing residues #581–590 of domain 4 of HER2 was modeled, since atomic coordinates of this region are missing in the crystal structure of Cho et al. Prior experience in loop modeling, both in our laboratory as well as elsewhere, has shown that *ab initio* modeling of loops of 10 residues in length may be done with reasonable reliability [12].

We performed rigid body macromolecular docking with these two structures using ZDOCK [13] as described in Methods. This suite of algorithms has performed quite well in the periodically held Critical Assessment of Prediction of Interactions (CAPRI) experiments [14]. Although we performed some limited energy minimization of the final structural model, this approach to modeling the complex excludes the possibility that significant conformational changes (with respect to the *apo* structures) may occur in either the HER2 or the G3 structure or both. Exclusion of such changes is a safe assumption to make for the following reasons: neither the X-ray crystal structure of trastuzumab bound to HER2 nor that of the bispecific antibody bH1 bound to HER2 [15] showed any significant conformational changes in domain 4 of HER2 due to the complexation. Furthermore, an analysis [16] of a number of X-ray crystal structures of DARPins bound to different proteins did not show any significant conformational changes in the DARPin structure. The grid size that we used in the docking translates to a grid spacing of 1.2 Å, which is a sufficiently fine resolution for the docking, and at the same time implicitly allows for some conformational ‘flexibility’ during the docking.

Numerous protein-protein docking studies over the past few years, including the CAPRI experiments, have shown that more accurate results are produced by using existing structural biological and biochemical experimental information to guide and filter the computational results [17]. Experimentally determined structures of complexes of DARPins with other proteins show that the concave face of the DARPin structure is used in binding the target [16]. This is consistent with expectations from the design [6] as this face is randomized and amino acids mediating tight binding are selected by ribosome display or phage display [7], [18]. In DARPins, the "constant" convex face, distal to the randomized and selected concave face, is characterized by the presence of a number of acidic amino acid residues, i.e. Glu 61, Glu 64, Glu 97, Asp 127, and Glu 130. Hence, in our computational docking, we required that none of these acidic residues would be present in the interacting surface of the ZDOCK docking solutions, i.e. such docked solutions were ‘blocked out’. The top 2000 docked solutions produced by this protocol were then scored and re-ranked by the secondary scoring function ZRANK [19]. The top ranked 20 solutions from this scoring step were selected for further analysis. This analysis consisted of diverse computational metrics as well as visual examination.

During the formation of a complex between two proteins, it can be reasonably expected that favorable interactions between the two molecules would be maximized, i.e. the thermodynamically most stable complex would be that with the optimum interaction energy. In protein-protein interactions this is largely achieved by maximizing the shape complementarity, and hence maximizing favorable van der Waals interactions between the two proteins. For this reason, we chose the total interaction energy (IE) and shape complementarity (Sc) between the two proteins as the primary metrics to select the ‘best’ solution from the top 20 ZRANKed solutions. The empirical force field FoldX [20] was used to evaluate IE. The FoldX force field contains parameters derived from experimental data, and had been developed for the purpose of evaluating the effects of mutations on protein and protein-protein complex stability in a rapid and accurate manner. We used the metric developed by Lawrence and Colman [21] to measure Sc at the interface of each of the top ranked solutions. This metric is easy to compute and was found by the developers to distinguish between different classes of protein-protein complexes. Table 1 lists the computed IE and Sc values for each of the top ranked 20 solutions.

**Table 1:** Top ZRANKed docking solutions and their computed metrics shape complementarity (Sc) and interaction energy (IE).

ZRANK #	ZDOCKsolution #	Sc	IE (kcal mol^−1^)
1	1698	0.425	−6.35
2	1	0.704	−15.40
3	566	0.663	−8.51
**4**	**45** a	**0.748**	−**19.37**
5	431	0.668	−3.61
6	20	0.673	−14.29
7	1094	0.414	−3.72
8	41	0.672	−13.46
9	380	0.658	−10.42
10	136	0.710	−13.33
11	240	0.400	−0.99
12	88	0.444	−10.98
13	21	0.520	−15.42
14	24	0.557	−13.65
15	1235	0.492	−8.29
16	268	0.386	−3.19
17	6	0.610	−15.75
18	195	0.550	−18.66
19	491	0.365	−6.15
20	8	0.529	−12.72

a This ZDOCK solution was selected as the optimal or preferred solution for the structural model of the G3 - HER2 complex.

On the basis of these metrics, we selected ZDOCK solution #45 as the optimal solution since it had both the highest shape complementarity (0.748) as well as the highest interaction energy (−19.37 kcal mol**^−^** ^1^) between the two protein components. While solutions #1 and #136 also have Sc greater than 0.7, visualizing these two solutions made it apparent that G3 in these instances was binding to the far C-terminal region of HER2 domain 4, such that their orientation made clashes with the cell membrane likely. We also considered the possibility of solution #195, which had the second highest interaction energy of −18.66 kcal mol**^−^** ^1^.

Two more metrics were considered in making the final decision: One was RPScore [22], which uses an empirically derived (from statistical analysis of non-homologous interfaces in the Protein Data Bank) amino acid residue pair potential matrix and gives the likelihood of the occurrence of the given residue pairs across the interface. The other was EC, the electrostatic complementarity at the interface [23] of the complex, computed by solving the linear Poisson-Boltzmann equation at the interface surfaces. These calculations gave a RPScore value of +2.40 and an EC (Pearson) value of +0.35 for the solution #45 while for solution #195 these values were +1.80 and +0.29, respectively. These additional metrics confirmed our selection of solution #45 as the preferred solution of the structure of the complex. (We note here that using EC as a metric for filtering docked solutions in general is impracticable as this calculation is very compute-intensive in nature). This structure was then further refined to the final model (see Text S1 for the coordinates of this model in Protein Data Bank format) by performing a limited amount of constrained energy minimization as described in Methods.

### Analysis of the Structural Model

**Figure 1:**
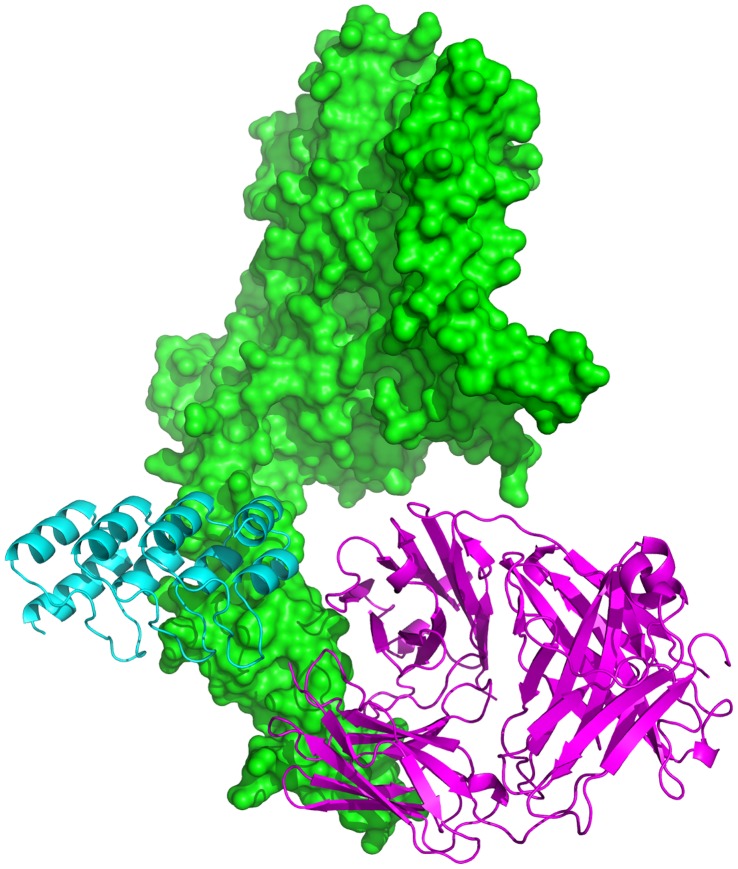
Structural model of the DARPin G3 (cyan) in complex with the human epidermal growth factor receptor 2 HER2 (green). In magenta the structure of trastuzumab Fab bound to HER2 is shown superimposed on the G3-HER2 complex.

The resultant three-dimensional atomic structure of the complete (all four domains) HER2 ectodomain – G3 complex is shown in Figure 1 in a molecular surface representation. (This was obtained by superimposing our computational structural model with the HER2 crystal structure of Cho et al. [4]). Figure 1 also depicts the structure of trastuzumab bound to HER2 (as given by the X-ray crystal structure of Cho et al. [4]) superimposed on the structural model of the complex. While the HER2 epitopes for the DARPin G3 and trastuzumab are adjacent and very close to each other, they do not overlap, in agreement with the experimental observation that trastuzumab and G3 binding to HER2 are not competitive with each other. It can also be seen that G3 does not make any contacts with HER2 domains 1–3 (in agreement with experimental observations), despite the fact that the latter were not considered at all by our modeling of the complex.

**Figure 2:**
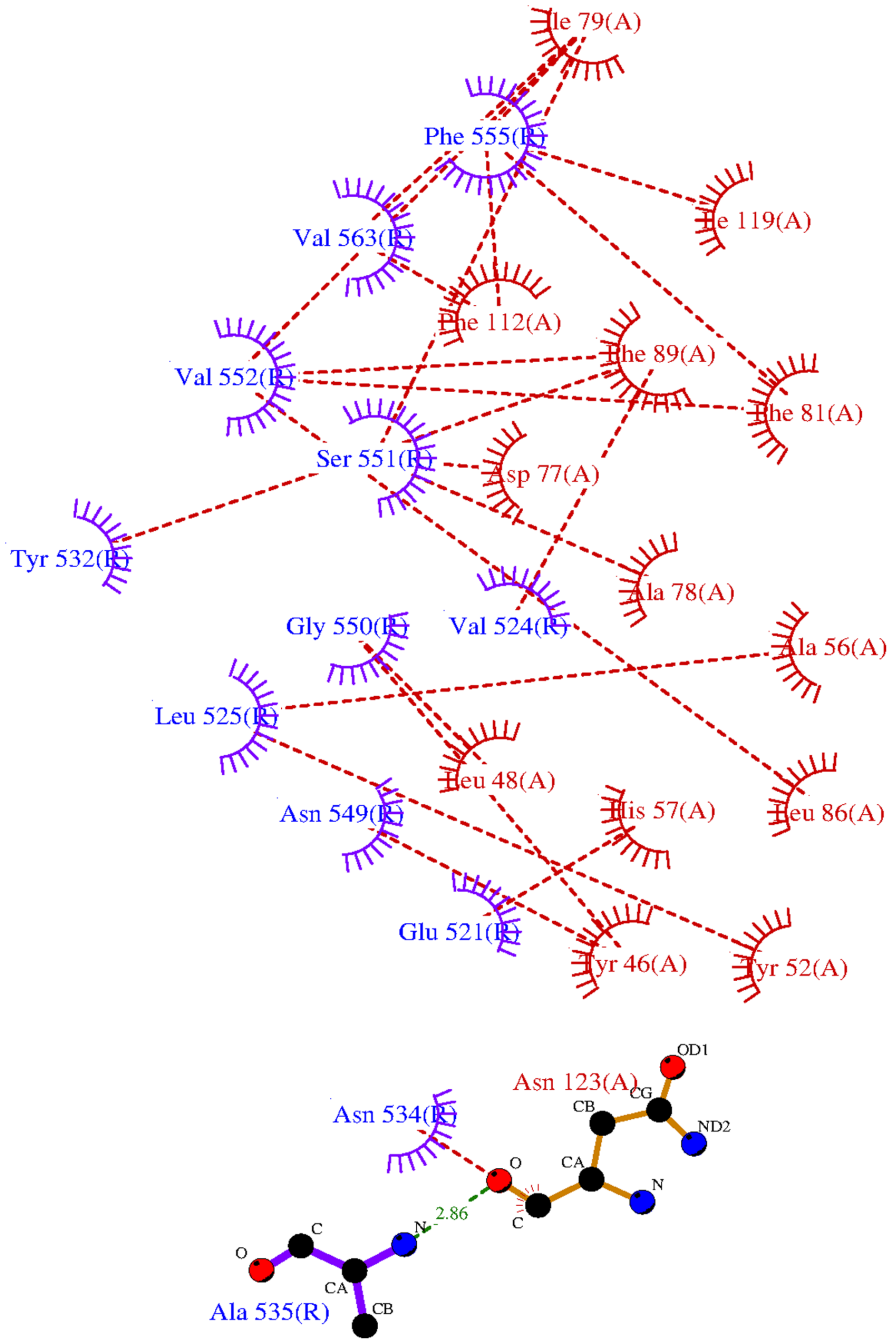
LigPlot diagram of all G3 (red) - HER2 (blue) residue interactions in the model.

The interface between HER2 domain 4 and G3 buries 961.8 Å^2^ of total surface area. Figure 2 depicts the interactions between HER2 and G3 present at the interface in a LigPlot figure [24]. With the exception of a backbone hydrogen bond between Ala 535 of HER2 and Asn 123 of G3, all the interactions are of van der Waals in nature. The largest number of interactions across the interface are by the HER2 amino acid residues Phe 555 (with G3 amino acid residues Ile 79, Phe 81, Phe 112, and Ile 119), Val 552 (with G3 residues Phe 81, Leu 86, and Phe 89), Ser 551 (with G3 residues Asp 77, Ala 78, and Ile 79), Val 563 (with G3 residues Ile 79 and Phe 112), Gly 550 (with G3 residues Tyr 46 and Leu 48), and Leu 525 (with G3 residues Tyr 52 and Ala 56). We note that of the interacting residues depicted in Figure 2, a relatively large number of residues on the part of G3, and a relatively small number of residues on the part of HER2, are aromatic. This characteristic of DARPin-antigen complexes has been commented on previously [6], [7], [16], and especially exposed Tyr residues are well-known for facilitating protein-protein interactions in antibodies and other complexes [25]. Five of the G3 interacting residues are from the first ankyrin repeat, six residues are from the second repeat, and three residues are from the C-cap region while none are from the N-cap region. The interacting G3 amino acid residues Tyr 46, Leu 48, Ala 56, His 57, Ala 78, Ile 79, Phe 81, and Phe 89 are all at randomized positions on the DARPin.

### Validation of the Structural Model

To validate our structural model of the HER2-G3 complex we mutated HER2 amino acid residues at the interface predicted by the model structure to make important interactions and stabilize the complex. We chose to mutate to alanine the HER2 amino acid residues Leu 525, Ser 551, Val 552, and Phe 555. According to our structural model each of these residues makes interactions with several G3 amino acids at the interface which are primarily van der Waals in nature. Hence mutating these residues to alanine would be expected to have a detrimental effect on G3-binding. These expectations were further supported by analyzing the complex structural model with the DrugScore^PPI^ [26] web server, which predicted that the Leu 525→Ala, Ser 551→Ala, Val 552→Ala, and Phe 555→Ala mutations should decrease the binding free energy by 0.87, 0.6, 1.92, and 0.77 kcal mol**^−^** ^1^, respectively. DrugScore^PPI^ is a knowledge-based scoring function for computational alanine scanning, derived from experimental structures of complexes and alanine scanning results. Finally, we utilized PROSA [27] to estimate whether the proposed mutations in the HER2 structure were stabilizing or destabilizing. PROSA is most frequently used to assess the quality of structural models of proteins. The computation using the knowledge-based potentials in PROSA, derived from 1352 high-resolution X-ray structures of proteins, concluded that the mutations Leu 525→Ala and Phe 555→Ala should be stabilizing to the HER2 protein structure while the mutations Ser 551→Ala and Val 552→Ala would not cause any significant changes to the stability. Since none of these amino acid residues are part of the epitope for trastuzumab, we anticipated that the introduction of alanine mutations at each position should not affect trastuzumab binding to the mutant HER2 isoforms.

Site-directed mutagenesis was used to introduce the selected mutations into a mammalian expression vector encoding residues 1–623 of the mature extracellular domain of HER2 and incorporating a C-terminal Flag tag. The introduction of the mutations had no effect on the expression yield and secretion of soluble HER2, established by western blotting using an anti-Flag monoclonal antibody on immune blotted cell culture supernatants derived from HEK-293 T cells transiently transfected with plasmid vectors encoding either wild-type or mutant HER2 isoforms (data not shown). Purified recombinant HER2 was isolated from supernatants of scaled-up transiently transfected cultures of HEK-293F cells by immunoaffinity chromatography.

### Surface Plasmon Resonance

**Figure 3:**
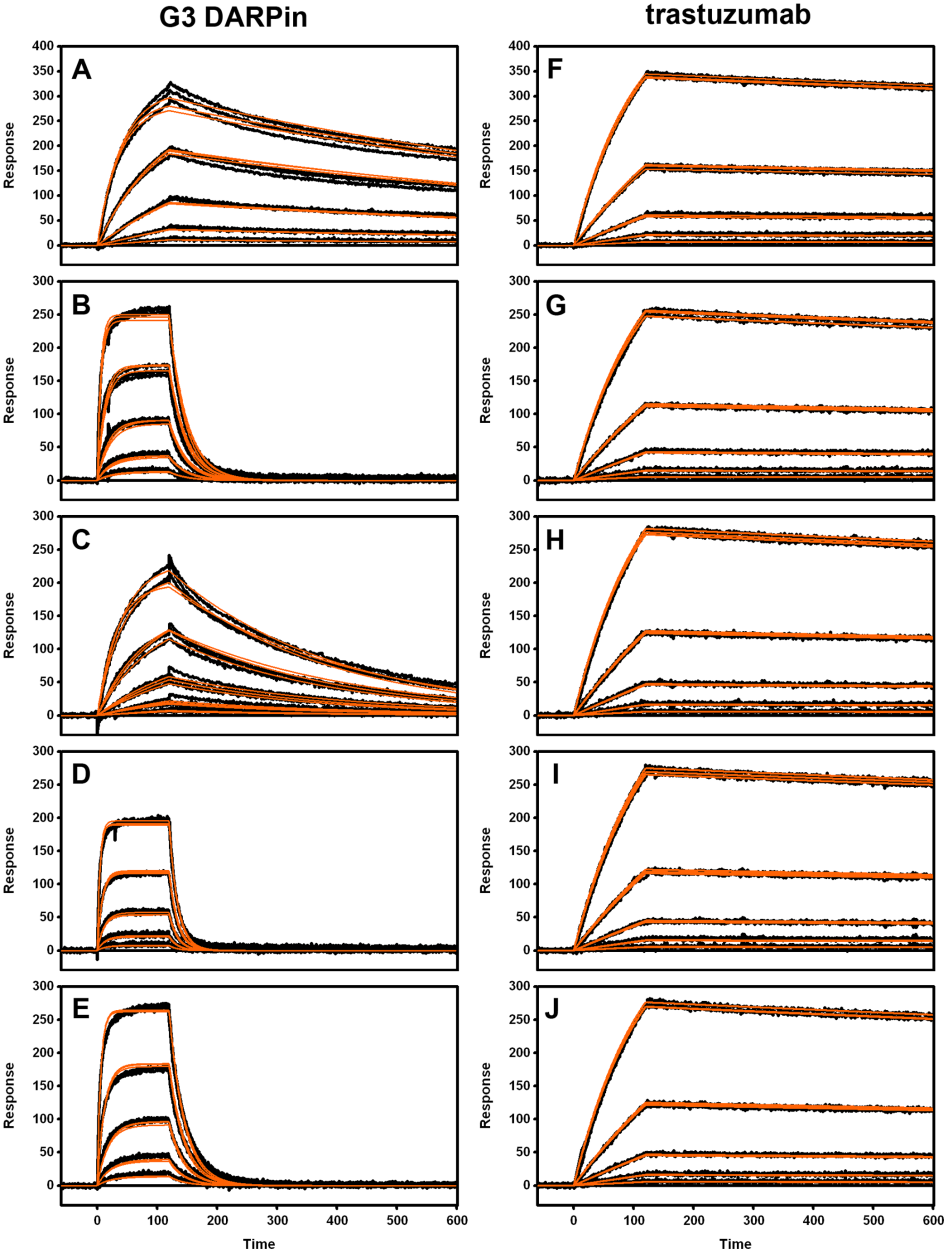
SPR binding sensorgrams for the interaction of HER2 wild type and mutant proteins with immobilized DARPin G3 (left panels) and trastuzumab (right panels). Injected analyte (HER2) protein construct: wild type (A and F), Leu 525→Ala (B and G), Ser 551→Ala (C and H), Val 552→Ala (D and I) and Phe 555→Ala (E and J). Typically, injected HER 2 protein concentrations were diluted three-fold in running buffer from 81 nM to 1 nM except in B, D and E where the concentration series were diluted from 729 nM down to 9 nM. Overlayed triplicate binding responses are shown (black lines). Binding data were globally fit to a simple 1∶1 interaction model (orange lines).

Surface Plasmon Resonance (SPR) was utilized for the kinetic interaction analyses of the HER2 wild type and mutant constructs. A concentration series of each HER2 mutant was injected over chip surfaces coated with either DARPin G3 or trastuzumab. Binding sensorgrams shown in Figure 3 and the corresponding binding rate parameters and overall affinity estimates listed in Table 2 clearly indicated that none of the introduced alanine mutations affected the binding of HER2 to immobilized trastuzumab. This provided further evidence that none of these mutations compromised structural integrity of the HER2 protein.

**Table 2:** SPR binding parameters for HER2 constructs binding to the DARPin G3 and trastuzumab.

	DARPin G3			
HER2 protein	k_a_×10^5^ (M^−1^s^−1^)	k_d_×10^−3^ (s^−1^)	K_D_ (nM)	fold difference in K_D_
wild-type^a^	3.22±0.20	1.07±0.01	3.3±0.2	1.0
Leu525-Ala	1.49±0.03	39.3±4.2	265±30	79.7
Ser551-Ala	2.85±0.59	3.5±2.1	12.8±3.1	3.8
Val552-Ala	1.69±0.16	64.0±3.7	380±39	114.4
Phe555-Ala	1.84±0.09	38.4±1.0	210±15	63.2
	**trastuzumab**			
**HER2 protein**	**k_a_×10^5^ (M^−1^s^−1^)**	**k_d_×10^−4^ (s^−1^)**	**K_D_ (nM)**	**fold difference in K_D_**
wild-type	1.19±0.09	1.56±0.03	1.31±0.13	1.0
Leu525-Ala	1.04±0.01	1.32±0.05	1.26±0.03	1.0
Ser551-Ala	1.07±0.03	1.47±0.01	1.38±0.04	1.0
Val552-Ala	0.96±0.02	1.28±0.08	1.33±0.06	1.0
Phe555-Ala	1.06±0.01	1.48±0.02	1.39±0.03	1.1

Note 1: The values given are the average values for three separate measurements and the uncertainties represent one standard deviation.

a Note 2: About 30-fold higher affinities are obtained when avoiding random amine coupling of this very small DARPin [9,36], (Nagy-Davidescu and Plückthun, unpublished).

DARPin G3 has been analyzed in SPR using both BIAcore [9] and ProteOn instruments (Nagy-Davidescu and Plückthun, unpublished). In many repeated measurements K_D_ values of 90–100 pM were obtained under all conditions, and this is consistent with measurements on cells as well [1]. For these previous SPR measurements, HER2 was coupled to the sensor, since amine coupling of the DARPin may interfere with the HER2 interaction of this very small protein. Indeed, a 30-fold lower affinity was observed here when amine-coupling the wild type DARPin G3 (Table 2). However, since only relative affinities of HER2 mutants were needed, this can still be used for comparisons.

More importantly, when compared with HER2 wild-type, estimated binding parameters for interactions of the HER2 mutants with DARPin G3 showed very clear differences. Thus, the Val 552→Ala mutation generated the most significant difference of the four mutant constructs tested with the measured affinity being more than 100-fold weaker. Similarly, Leu 525→Ala and Phe 555→Ala were also shown to significantly affect the binding to G3 DARPin resulting in an 80-fold and 63-fold reduction in affinity, respectively. The Ser 551→Ala mutation proved to change the binding least, generating only a 4-fold reduction in affinity. In all four cases the dissociation rate constant (k_d_) was most significantly affected and corresponded with the overall affinity. In contrast, the association rate constant (k_a_) was little affected, and it typically falls in a narrow window for protein-protein interactions [28] as it is mainly influenced by translational and rotational diffusion, which was little affected by the mutations.

### Structural Comparison with the X-ray Crystallographic Structure

During the preparation of this manuscript the X-ray crystallographic structure of the complex between H10-2-G3 and a construct of HER2 domain 4 was solved at a resolution of 2.65 Å (C. Jost et al., submitted). After obtaining the coordinates of the crystal structure (PDB id: 4HRN), we compared the structure with that of the computational model.

**Figure 4:**
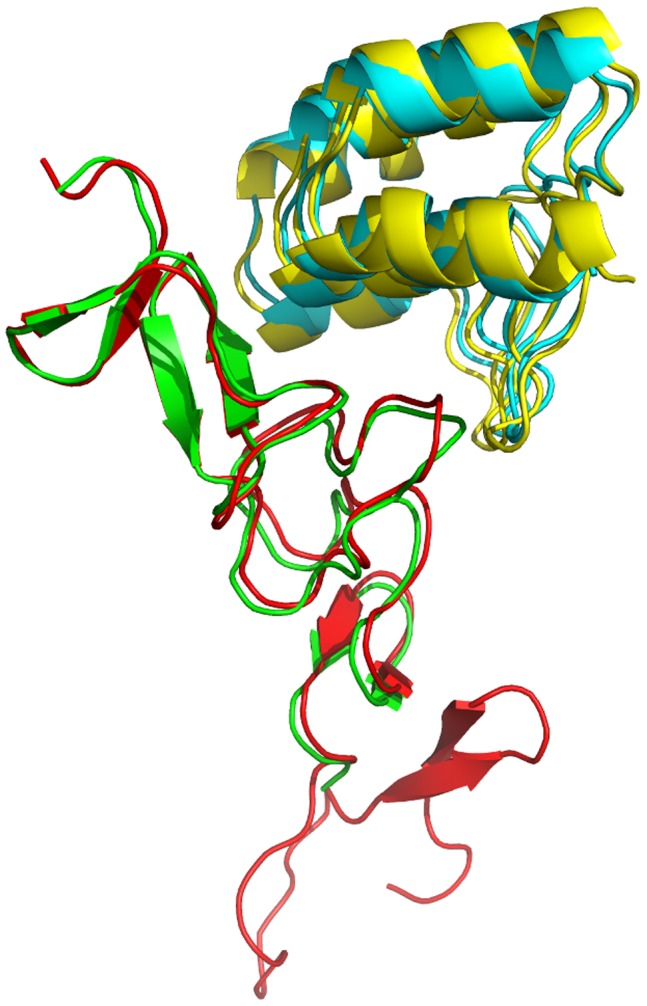
The computational model of the complex superimposed on the B and C chains of the X-ray crystal structure (BCxray). The G3 chains of the model and BCxray are in yellow and cyan, respectively, while the HER2 chains of the model and BCxray are shown in red and green, respectively.

The X-ray crystal structure has two complex molecules in the asymmetric unit with each consisting of the complex between the DARPin H10-2-G3 (chain A with residues #13–133 and chain B with residues #13–135) and HER2 (chain C with residues #509–579, and chain D with residues #509–578). Thus we shall refer to the 2 complexes in the crystal structure as ‘ADxray’ and ‘BCxray’. Superimposing the backbone atoms of the G3 residues #13–133 and HER2 residues #509–578 of ADxray and BCxray on the corresponding atoms of the computational model (hereafter referred to as the ‘model’) gives a root mean square deviation (rmsd) value of 1.14 Å between ADxray and model and a value of 0.84 Å between BCxray and model. In the more detailed comparisons discussed below we shall restrict ourselves to the comparison between the model and BCxray. Figure 4 depicts the model superimposed (as described above) on BCxray.

The CAPRI experiments [14] use a number of standard metrics to assess the structural models that are submitted. These include f_nat_ (the fraction of native contacts present in the model), f_non-nat_ (the fraction of non-native contacts), L-RMSD (the ligand rmsd after the receptor, defined as the larger of the two proteins, is superimposed), and I-RMSD (the rmsd for the interface residues). The interface in BCxray (analyzed with PDBsum [29]) consists of amino acid residues Tyr 46, Leu 48, Tyr 52, Ala 56, His 57, Asp 77, Ala 78, Ile 79, Phe 81, Phe 89, Ile 90, His 92, Phe 112, Gly 122, Asn 123, and Gly 124 of the DARPin, and residues Phe 512, Glu 521, Leu 525, Gln 526, Tyr 532, Val 533, Asn 534, Ala 535, Asp 549, Gly 550, Ser 551, Cys 554, Phe 555, and Val 563 of HER2. In the complex BCxray, these interface residues contribute to a total of 26 residue-residue contacts, out of which 24 are present in the model. This gives a f_nat_ value of 0.92 for the model. The f_non-nat_ value for the model is 0.47. The I-RMSD value (i.e. for the interface residues) is 0.92 Å, while the L-RMSD value for the model is 1.84 Å. (We note here that in deriving the L-RMSD value we followed the CAPRI convention and treated the HER2 chains as the ‘ligand’. If instead, we take HER2 as the ‘receptor’ the ‘L-RMSD’ value is 1.38 Å.) The CAPRI assessment classifies models with f_nat_ ≥0.5 *and* (L_RMSD ≤1.0 *or* I_RMSD ≤1.0) as ‘highly accurate’ models. Accordingly, this model can be classed as ‘highly accurate’. This however, should also be tempered by possible ‘false positive’ interactions indicated by the f_non-nat_ value, likely caused by the energy minimization in the final refinement.

In summary, we have, in this work, constructed a three-dimensional atomic model of the complex between HER2 and the DARPin G3 using computational methodologies and metrics. It utilized the *apo* crystal structures of HER2 and the DARPin as well as information on the binding face of the DARPin. The model was tested with HER2 mutants selected from the structurally characterized interface of the complex, showing reduced binding to G3. The work described here not only resulted in a structural model of reasonably good accuracy for the interaction between HER2 and G3, but also provided a validated examination of the capabilities of the computational methodologies.

## Materials and Methods

### Modeling

#### Preparation of the starting structures

The starting structures for the protein-protein docking were derived from the X-ray crystal structure of HER2 (i.e. ErbB2) in complex with trastuzumab [4] (PDB id 1N8Z), and the X-ray crystal structure of the DARPin H10-2-G3 [9] (PDB id 2JAB), referred to as ‘G3’. Chain A (residues # 12–135) of the G3 structure was used in the modeling. Only domain 4 of HER2 was utilized in the docking calculations. In this domain, the atomic coordinates of the residues # 581–590 are missing due to disorder. Hence, prior to the macromolecular docking, the structure of this loop was modeled using Modeller v. 7.6 [30]. From the 20 loops constructed for this region, model #12, the one with the lowest value of –log_e_PDF (where PDF is the molecular probability density function), was selected as the optimal model.

#### Macromolecular docking

For the docking of these two protein structures we used the macromolecular docking program ZDOCK v. 3.0.1 [13]. ZDOCK is a grid-based rigid body docking algorithm, which discretizes the two proteins (labeled the “receptor” and the “ligand”) into grids, and performs global scans of the rotational and translational space of the ligand with respect to the receptor, with each relative orientation scored by a shape complementarity function. In our docking, we designated domain 4 of HER2 as the “receptor” and the DARPin G3 as the “ligand”. Our grid size of 128 yielded a grid spacing of 1.2 Å, while the rotational sampling was done with an interval of 6 degrees. The search space for ZDOCK was effectively reduced by requiring that the docking solutions excluded the convex face of G3. This was done by specifying that the amino acid residues Glu 61, Glu 64, Glu 97, Asp 127, and Glu 130 that are on the convex face of G3 be absent from the interacting surface of the ZDOCK docking solutions, i.e. these residues were ‘blocked out’ during the docking.

The top 2000 docked solutions produced by this protocol were then scored and re-ranked by the secondary scoring function ZRANK [19]. This function is a linear weighted sum of van der Waals, Coulomb, and desolvation energy terms, where the optimal weights had been obtained by training the function on a benchmark set of protein-protein complexes. The top ranked 20 solutions from this step were selected for further analysis.

We used the total interaction energy (IE) and shape complementarity (Sc) between the two proteins as the primary metrics to select the ‘best’ solution from the top 20 ZRANKed solutions. The empirical force field FoldX [20] was used to evaluate the interaction IE. This force field free energy consists of a linear combination of terms due to van der Waals energy, solvation, hydrogen bonding, Coulomb electrostatics, and entropy changes. We used the metric developed by Lawrence and Colman [21] to measure the shape complementarity Sc at the interface of each of the top ranked solutions.

We also used the computed metrics RPScore and EC in the final choice of the optimal solution. RPScore [22] uses an empirically derived (from statistical analysis of non-homologous interfaces in the Protein Data Bank) amino acid residue pair potential matrix and gives the likelihood of the occurrence of the given residue pairs across the interface. EC, the electrostatic complementarity at the interface [23] of the complex, was computed by solving the linear Poisson-Boltzmann equation at the interface surfaces.

#### Refinement and analysis of the model

Prior to the structural characterization of the final optimal solution, the selected top solution of the complex structure was further refined by performing a limited amount of constrained energy minimization with the program Discover v. 2.98 within InsightII v.2005 (Accelrys, Inc.). After adding hydrogen atoms to the structure, the energy was first minimized with 500 steps of steepest descents, holding the backbone of HER2 domain 4 fixed and tethering the backbone of G3 with a force constant of 10.0 kcal Å**^−^** ^2^. Next, 100 steps of steepest descents were performed by tethering the backbone of HER2 domain 4 with a force constant of 5.0 kcal Å**^−^** ^2^. The CVFF force field was used with a distance-dependant dielectric and no Morse or cross terms in the application of the force field. The resultant structure of the complex was analyzed and the interface between HER2 and G3 was characterized using LigPlot [24].

### Molecular and Cell Biology

The mammalian expression vector pME18s.HER2-623, encoding the natural signal peptide and residues 1–623 of the extracellular domain of HER2, and incorporating a C-terminal Flag tag, was used as a template for site-directed mutagenesis. Selected residues were mutated using the QuikChange site-directed mutagenesis protocol (Stratagene) and the mutagenic primer pairs listed in Table S1. The successful incorporation of mutations was confirmed by DNA sequencing.

The culture and transient transfection of human 293 T fibroblasts was performed as previously described [31]. The successful biosynthesis and secretion of wild-type and mutant HER2 isoforms was established by western blotting of culture supernatants using an anti-Flag tag-specific monoclonal antibody. For the purification of recombinant HER2 ectodomain, suspension-adapted cultures (200 ml) of Freestyle 293-F cells (Invitrogen) grown in Freestyle 293 Expression Medium were transiently transfected with plasmid DNA using polyethylenimine (PEI; [32]). Following culture for 7–9 days, supernatants were harvested and recombinant HER2 purified by anti-Flag immunoaffinity chromatography [33].

### Surface Plasmon Resonance

All SPR experiments were performed at 25°C using Bio-Rad’s ProteOn XPR36 array biosensor [34]. A standard amine-coupling protocol was employed to immobilize G3 DARPin on a GLC chip surface in 1× HBS-P buffer (10 mM HEPES, 150 mM NaCl, 0.05% (v/v) Tween 20) at a constant flow rate of 30 μl/min. Briefly, with instrumental fluidics oriented in the “vertical” direction, a single lane on the chip surface was activated by a 5-min injection of a freshly prepared mixture consisting of 2.5 mM sulfo-NHS and 10 mM EDC. G3 DARPin solution (15 μg/ml in 10 mM sodium acetate, pH 5.0) was then injected for 5 min and any residual reactive sites deactivated by a final 5-min injection of 1 M ethanolamine (pH 8.5). Approximately 1,200 RU (1 RU  = 1 pg of protein/mm^2^) of G3 DARPin was coupled using this method. Trastuzumab was captured onto the SPR sensor chip surface using a previously described Protein G’ capture method [35]. Briefly, Protein G’ (Sigma-Aldrich) was coupled in a single lane on a GLC chip at 2,200 RU using an identical amine coupling method described for G3 DARPin except that Protein G’ was injected at 50 μg/ml in 10 mM sodium acetate, pH 4.0. A single injection of trastuzumab at (5 μg/ml, 100 μl/min for 30 sec) in the vertical orientation resulted in a consistent capture of approximately 1,300 RU of protein across entire Protein G’ lane. No significant dissociation (drift) of trastuzumab from the Protein G’ surface was observed (drift ≤1 RU/600 sec).

The ‘One-Shot Kinetics’ approach of Bravman et al. [34] was utilized for binding analyses of HER2 proteins. Binding assays were performed in 1× HBS-EBP+ buffer (10 mM HEPES, 150 mM NaCl, 3 mM EDTA, 0.1% [w/v] BSA; 0.05% [v/v] Tween 20) with the instrumental fluidics oriented in “horizontal” direction. Six concentrations of HER2 (including “zero buffer blank”) were injected simultaneously over amine-coupled G3 DARPin or Protein G’-captured trastuzumab at 30 μL/min for 120 sec. The dissociation phase was monitored until all of the bound HER2 protein had dissociated from the DARPin-coupled surface. This was possible, since random amine coupling of the DARPin lowered its known K_D_ of 90–100 pM about 30-fold to 3 nM (Table 2), while the reverse set-up leads to almost no dissociation. In case of the trastuzumab-HER2 complex, complete dissociation was not achievable in a practicable timeframe. Consequently, after each binding cycle, the Protein G’ surface was regenerated in the vertical direction with a single injection of 10 mM glycine pH 1.5 (100 μl/min, 18 sec). Trastuzumab was recaptured for any subsequent binding cycles.

All SPR binding data were processed using the Scrubber-Pro software package (www.biologic.com.au). To determine the kinetic rate constants (k_a_ and k_d_) of the binding interactions, binding data were fit globally to a 1∶1 interaction model and the ratio of these rate constants (k_d_/k_a_) yielded the value for the equilibrium dissociation constant (K_D_).

### Structural Analysis

Structural superimposition and comparison was done with ProFit v. 2.5.3 (A.C.R. Martin, and C.T. Porter, www.bioinf.org.ac.uk/software/profit/) while the interface analysis was performed with PDBsum [29] (www.ebi.ac.uk/pdbsum). Figures 1 and 4 were prepared with PyMol v. 1.5.0.4 (Schrödinger, LLC.).

## Supporting Information

Table S1 Oligonucleotide pairs for site-directed mutagenesis of HER2.
(DOCX)

Text S1 **Coordinates of the computational model for the G3-HER2 complex in PDB format.**
(DOCX)
