# Supplementary material for: Structural Model for the Interaction of a Designed Ankyrin Repeat Protein with the Human Epidermal Growth Factor Receptor 2
Source: PLoS One. 2013 Mar 19;8(3):e59163. doi: 10.1371/journal.pone.0059163 (PMC3602593; doi:10.1371/journal.pone.0059163)
Supplement: Table S1 — Oligonucleotide pairs for site-directed mutagenesis of HER2. (DOCX) [file pone.0059163.s001.docx]

**Table S1** Oligonucleotide pairs for site-directed mutagenesis of HER2. Changes from the wild-type sequence are underlined.

1. ErbB2 mutant L525A:

Forward 5’ AGGAATGCCGAGTAGCGCAGGGGCTCCCCA 3’

Reverse 5’ TGGGGAGCCCC GCGCTACTCGGCATTCCT 3’

1. ErbB2 mutant S551A:

Forward 5’ CCCCAGAATGGCGCAGTGACCTGTTT 3’

Reverse 5’ AAACAGGTCACTGCGCCATTCTGGGG 3’

1. ErbB2 mutant V552A:

Forward 5’ CAGAATGGCTCAGCGACCTGTTTT GG 3’

Reverse 5’ CCAAAACAGGTCGCTGAGCCATTCTG 3’

1. ErbB2 mutant F555A:

Forward 5’ CTCAGTGACCTGTGCTGGACCGGAGGCTG 3’

Reverse 5’ CAGCCTCCGGTCCAGCACAGGTCACTGAG 3’
