# Supplementary material for: Structural Model for the Interaction of a Designed Ankyrin Repeat Protein with the Human Epidermal Growth Factor Receptor 2
Source: PLoS One. 2013 Mar 19;8(3):e59163. doi: 10.1371/journal.pone.0059163 (PMC3602593; doi:10.1371/journal.pone.0059163)
Supplement: Text S1 — Coordinates of the computational model for the G3-HER2 complex in PDB format. (DOCX) [file pone.0059163.s002.docx]

REMARK cmplx4501_cor_noH.pdb

REMARK Model of complex between HER2 domain 4 (chain 'R', residues: 508-607) and DARPin G3 (chain 'A')

REMARK Vidana C. Epa, March 2011.

ATOM 1 N ASN R 508 19.349 88.707 105.104 1.00 1.00 N

ATOM 2 CA ASN R 508 20.292 89.490 104.263 1.00 1.00 C

ATOM 3 CB ASN R 508 19.529 90.551 103.424 1.00 1.00 C

ATOM 4 CG ASN R 508 18.988 89.980 102.114 1.00 1.00 C

ATOM 5 OD1 ASN R 508 19.747 89.613 101.228 1.00 1.00 O

ATOM 6 ND2 ASN R 508 17.697 89.838 101.954 1.00 1.00 N

ATOM 7 C ASN R 508 21.371 90.133 105.173 1.00 1.00 C

ATOM 8 O ASN R 508 21.042 90.754 106.188 1.00 1.00 O

ATOM 9 N CYS R 509 22.653 90.000 104.791 1.00 1.00 N

ATOM 10 CA CYS R 509 23.751 90.740 105.453 1.00 1.00 C

ATOM 11 CB CYS R 509 25.068 90.132 104.941 1.00 1.00 C

ATOM 12 SG CYS R 509 26.485 90.893 105.759 1.00 1.00 S

ATOM 13 C CYS R 509 23.651 92.266 105.160 1.00 1.00 C

ATOM 14 O CYS R 509 23.529 92.691 104.005 1.00 1.00 O

ATOM 15 N SER R 510 23.653 93.079 106.224 1.00 1.00 N

ATOM 16 CA SER R 510 23.554 94.561 106.090 1.00 1.00 C

ATOM 17 CB SER R 510 23.081 95.115 107.454 1.00 1.00 C

ATOM 18 OG SER R 510 24.098 95.046 108.456 1.00 1.00 O

ATOM 19 C SER R 510 24.818 95.302 105.544 1.00 1.00 C

ATOM 20 O SER R 510 24.690 96.389 104.977 1.00 1.00 O

ATOM 21 N GLN R 511 26.011 94.717 105.729 1.00 1.00 N

ATOM 22 CA GLN R 511 27.297 95.291 105.277 1.00 1.00 C

ATOM 23 CB GLN R 511 28.157 95.567 106.556 1.00 1.00 C

ATOM 24 CG GLN R 511 27.639 96.592 107.590 1.00 1.00 C

ATOM 25 CD GLN R 511 27.417 98.019 107.083 1.00 1.00 C

ATOM 26 OE1 GLN R 511 27.852 98.445 106.018 1.00 1.00 O

ATOM 27 NE2 GLN R 511 26.713 98.808 107.852 1.00 1.00 N

ATOM 28 C GLN R 511 27.905 94.319 104.218 1.00 1.00 C

ATOM 29 O GLN R 511 27.473 94.304 103.060 1.00 1.00 O

ATOM 30 N PHE R 512 28.888 93.501 104.620 1.00 1.00 N

ATOM 31 CA PHE R 512 29.700 92.673 103.701 1.00 1.00 C

ATOM 32 CB PHE R 512 31.146 93.250 103.609 1.00 1.00 C

ATOM 33 CG PHE R 512 31.236 94.680 103.051 1.00 1.00 C

ATOM 34 CD1 PHE R 512 31.122 94.896 101.678 1.00 1.00 C

ATOM 35 CD2 PHE R 512 31.256 95.777 103.920 1.00 1.00 C

ATOM 36 CE1 PHE R 512 30.972 96.188 101.185 1.00 1.00 C

ATOM 37 CE2 PHE R 512 31.125 97.071 103.424 1.00 1.00 C

ATOM 38 CZ PHE R 512 30.968 97.274 102.056 1.00 1.00 C

ATOM 39 C PHE R 512 29.702 91.209 104.213 1.00 1.00 C

ATOM 40 O PHE R 512 29.591 90.933 105.410 1.00 1.00 O

ATOM 41 N LEU R 513 29.850 90.253 103.299 1.00 1.00 N

ATOM 42 CA LEU R 513 30.018 88.822 103.660 1.00 1.00 C

ATOM 43 CB LEU R 513 29.031 87.967 102.828 1.00 1.00 C

ATOM 44 CG LEU R 513 27.584 87.960 103.379 1.00 1.00 C

ATOM 45 CD1 LEU R 513 26.584 87.765 102.233 1.00 1.00 C

ATOM 46 CD2 LEU R 513 27.396 86.870 104.451 1.00 1.00 C

ATOM 47 C LEU R 513 31.477 88.400 103.397 1.00 1.00 C

ATOM 48 O LEU R 513 31.968 88.500 102.270 1.00 1.00 O

ATOM 49 N ARG R 514 32.157 87.867 104.415 1.00 1.00 N

ATOM 50 CA ARG R 514 33.405 87.100 104.194 1.00 1.00 C

ATOM 51 CB ARG R 514 34.519 87.698 105.062 1.00 1.00 C

ATOM 52 CG ARG R 514 35.896 87.014 104.844 1.00 1.00 C

ATOM 53 CD ARG R 514 37.108 87.914 105.129 1.00 1.00 C

ATOM 54 NE ARG R 514 37.057 88.454 106.519 1.00 1.00 N

ATOM 55 CZ ARG R 514 36.895 89.742 106.830 1.00 1.00 C

ATOM 56 NH1 ARG R 514 37.108 90.742 106.012 1.00 1.00 N

ATOM 57 NH2 ARG R 514 36.501 90.034 108.033 1.00 1.00 N

ATOM 58 C ARG R 514 33.112 85.603 104.460 1.00 1.00 C

ATOM 59 O ARG R 514 32.833 85.191 105.592 1.00 1.00 O

ATOM 60 N GLY R 515 33.114 84.804 103.380 1.00 1.00 N

ATOM 61 CA GLY R 515 32.557 83.432 103.416 1.00 1.00 C

ATOM 62 C GLY R 515 31.036 83.413 103.677 1.00 1.00 C

ATOM 63 O GLY R 515 30.242 83.909 102.876 1.00 1.00 O

ATOM 64 N GLN R 516 30.670 82.874 104.840 1.00 1.00 N

ATOM 65 CA GLN R 516 29.279 82.973 105.384 1.00 1.00 C

ATOM 66 CB GLN R 516 28.751 81.527 105.612 1.00 1.00 C

ATOM 67 CG GLN R 516 28.832 80.522 104.431 1.00 1.00 C

ATOM 68 CD GLN R 516 27.988 80.871 103.200 1.00 1.00 C

ATOM 69 OE1 GLN R 516 26.763 80.846 103.225 1.00 1.00 O

ATOM 70 NE2 GLN R 516 28.598 81.188 102.082 1.00 1.00 N

ATOM 71 C GLN R 516 29.150 83.828 106.695 1.00 1.00 C

ATOM 72 O GLN R 516 28.116 83.771 107.369 1.00 1.00 O

ATOM 73 N GLU R 517 30.169 84.632 107.050 1.00 1.00 N

ATOM 74 CA GLU R 517 30.135 85.533 108.215 1.00 1.00 C

ATOM 75 CB GLU R 517 31.515 85.482 108.924 1.00 1.00 C

ATOM 76 CG GLU R 517 31.452 85.836 110.432 1.00 1.00 C

ATOM 77 CD GLU R 517 32.757 86.352 111.021 1.00 1.00 C

ATOM 78 OE1 GLU R 517 33.784 85.644 110.952 1.00 1.00 O

ATOM 79 OE2 GLU R 517 32.755 87.475 111.565 1.00 1.00 O

ATOM 80 C GLU R 517 29.778 86.949 107.686 1.00 1.00 C

ATOM 81 O GLU R 517 30.480 87.520 106.838 1.00 1.00 O

ATOM 82 N CYS R 518 28.668 87.507 108.187 1.00 1.00 N

ATOM 83 CA CYS R 518 28.318 88.921 107.934 1.00 1.00 C

ATOM 84 CB CYS R 518 26.828 89.100 108.266 1.00 1.00 C

ATOM 85 SG CYS R 518 26.280 90.734 107.743 1.00 1.00 S

ATOM 86 C CYS R 518 29.246 89.849 108.773 1.00 1.00 C

ATOM 87 O CYS R 518 29.277 89.785 110.006 1.00 1.00 O

ATOM 88 N VAL R 519 30.059 90.640 108.072 1.00 1.00 N

ATOM 89 CA VAL R 519 31.178 91.413 108.669 1.00 1.00 C

ATOM 90 CB VAL R 519 32.588 90.919 108.180 1.00 1.00 C

ATOM 91 CG1 VAL R 519 32.884 89.475 108.636 1.00 1.00 C

ATOM 92 CG2 VAL R 519 32.873 91.059 106.666 1.00 1.00 C

ATOM 93 C VAL R 519 30.957 92.931 108.430 1.00 1.00 C

ATOM 94 O VAL R 519 30.350 93.367 107.443 1.00 1.00 O

ATOM 95 N GLU R 520 31.518 93.732 109.344 1.00 1.00 N

ATOM 96 CA GLU R 520 31.434 95.213 109.276 1.00 1.00 C

ATOM 97 CB GLU R 520 31.951 95.750 110.641 1.00 1.00 C

ATOM 98 CG GLU R 520 31.666 97.254 110.894 1.00 1.00 C

ATOM 99 CD GLU R 520 30.199 97.688 110.954 1.00 1.00 C

ATOM 100 OE1 GLU R 520 29.301 96.849 111.189 1.00 1.00 O

ATOM 101 OE2 GLU R 520 29.948 98.895 110.774 1.00 1.00 O

ATOM 102 C GLU R 520 32.134 95.843 108.032 1.00 1.00 C

ATOM 103 O GLU R 520 31.500 96.593 107.290 1.00 1.00 O

ATOM 104 N GLU R 521 33.412 95.496 107.805 1.00 1.00 N

ATOM 105 CA GLU R 521 34.181 95.945 106.625 1.00 1.00 C

ATOM 106 CB GLU R 521 35.141 97.111 107.010 1.00 1.00 C

ATOM 107 CG GLU R 521 34.455 98.440 107.429 1.00 1.00 C

ATOM 108 CD GLU R 521 33.520 99.086 106.414 1.00 1.00 C

ATOM 109 OE1 GLU R 521 33.702 98.912 105.189 1.00 1.00 O

ATOM 110 OE2 GLU R 521 32.603 99.803 106.858 1.00 1.00 O

ATOM 111 C GLU R 521 34.958 94.735 106.029 1.00 1.00 C

ATOM 112 O GLU R 521 35.502 93.864 106.729 1.00 1.00 O

ATOM 113 N CYS R 522 35.081 94.737 104.696 1.00 1.00 N

ATOM 114 CA CYS R 522 36.154 93.978 104.016 1.00 1.00 C

ATOM 115 CB CYS R 522 35.788 93.929 102.524 1.00 1.00 C

ATOM 116 SG CYS R 522 34.232 93.082 102.230 1.00 1.00 S

ATOM 117 C CYS R 522 37.552 94.632 104.250 1.00 1.00 C

ATOM 118 O CYS R 522 37.670 95.853 104.422 1.00 1.00 O

ATOM 119 N ARG R 523 38.629 93.829 104.228 1.00 1.00 N

ATOM 120 CA ARG R 523 40.021 94.347 104.403 1.00 1.00 C

ATOM 121 CB ARG R 523 40.930 93.184 104.871 1.00 1.00 C

ATOM 122 CG ARG R 523 40.564 92.559 106.236 1.00 1.00 C

ATOM 123 CD ARG R 523 41.485 91.377 106.559 1.00 1.00 C

ATOM 124 NE ARG R 523 40.884 90.517 107.613 1.00 1.00 N

ATOM 125 CZ ARG R 523 40.548 89.235 107.460 1.00 1.00 C

ATOM 126 NH1 ARG R 523 40.560 88.594 106.319 1.00 1.00 N

ATOM 127 NH2 ARG R 523 40.163 88.572 108.508 1.00 1.00 N

ATOM 128 C ARG R 523 40.572 95.053 103.120 1.00 1.00 C

ATOM 129 O ARG R 523 41.560 94.663 102.500 1.00 1.00 O

ATOM 130 N VAL R 524 39.901 96.138 102.748 1.00 1.00 N

ATOM 131 CA VAL R 524 40.160 96.905 101.500 1.00 1.00 C

ATOM 132 CB VAL R 524 38.785 97.519 101.055 1.00 1.00 C

ATOM 133 CG1 VAL R 524 38.923 98.494 99.881 1.00 1.00 C

ATOM 134 CG2 VAL R 524 37.765 96.434 100.645 1.00 1.00 C

ATOM 135 C VAL R 524 41.320 97.929 101.709 1.00 1.00 C

ATOM 136 O VAL R 524 42.284 97.962 100.940 1.00 1.00 O

ATOM 137 N LEU R 525 41.194 98.750 102.758 1.00 1.00 N

ATOM 138 CA LEU R 525 42.245 99.705 103.194 1.00 1.00 C

ATOM 139 CB LEU R 525 41.510 101.017 103.581 1.00 1.00 C

ATOM 140 CG LEU R 525 40.748 101.784 102.465 1.00 1.00 C

ATOM 141 CD1 LEU R 525 40.514 103.216 102.954 1.00 1.00 C

ATOM 142 CD2 LEU R 525 41.466 101.835 101.103 1.00 1.00 C

ATOM 143 C LEU R 525 43.170 99.234 104.361 1.00 1.00 C

ATOM 144 O LEU R 525 44.260 99.784 104.540 1.00 1.00 O

ATOM 145 N GLN R 526 42.720 98.278 105.183 1.00 1.00 N

ATOM 146 CA GLN R 526 43.386 97.866 106.441 1.00 1.00 C

ATOM 147 CB GLN R 526 42.693 98.555 107.647 1.00 1.00 C

ATOM 148 CG GLN R 526 42.873 100.091 107.761 1.00 1.00 C

ATOM 149 CD GLN R 526 42.065 100.759 108.879 1.00 1.00 C

ATOM 150 OE1 GLN R 526 42.599 101.436 109.748 1.00 1.00 O

ATOM 151 NE2 GLN R 526 40.760 100.608 108.898 1.00 1.00 N

ATOM 152 C GLN R 526 43.237 96.331 106.609 1.00 1.00 C

ATOM 153 O GLN R 526 42.309 95.704 106.090 1.00 1.00 O

ATOM 154 N GLY R 527 44.124 95.731 107.410 1.00 1.00 N

ATOM 155 CA GLY R 527 43.970 94.313 107.808 1.00 1.00 C

ATOM 156 C GLY R 527 44.898 93.350 107.056 1.00 1.00 C

ATOM 157 O GLY R 527 45.341 93.609 105.930 1.00 1.00 O

ATOM 158 N LEU R 528 45.179 92.238 107.746 1.00 1.00 N

ATOM 159 CA LEU R 528 46.335 91.350 107.450 1.00 1.00 C

ATOM 160 CB LEU R 528 46.418 90.292 108.591 1.00 1.00 C

ATOM 161 CG LEU R 528 46.833 90.746 110.014 1.00 1.00 C

ATOM 162 CD1 LEU R 528 46.641 89.572 110.992 1.00 1.00 C

ATOM 163 CD2 LEU R 528 48.292 91.228 110.074 1.00 1.00 C

ATOM 164 C LEU R 528 46.385 90.791 105.980 1.00 1.00 C

ATOM 165 O LEU R 528 47.219 91.315 105.232 1.00 1.00 O

ATOM 166 N PRO R 529 45.530 89.853 105.473 1.00 1.00 N

ATOM 167 CA PRO R 529 45.395 89.615 104.019 1.00 1.00 C

ATOM 168 CD PRO R 529 44.703 88.935 106.281 1.00 1.00 C

ATOM 169 CB PRO R 529 44.967 88.140 104.003 1.00 1.00 C

ATOM 170 CG PRO R 529 44.113 87.957 105.262 1.00 1.00 C

ATOM 171 C PRO R 529 44.367 90.600 103.389 1.00 1.00 C

ATOM 172 O PRO R 529 43.166 90.561 103.682 1.00 1.00 O

ATOM 173 N ARG R 530 44.857 91.490 102.517 1.00 1.00 N

ATOM 174 CA ARG R 530 43.989 92.466 101.812 1.00 1.00 C

ATOM 175 CB ARG R 530 44.859 93.489 101.038 1.00 1.00 C

ATOM 176 CG ARG R 530 45.672 94.463 101.911 1.00 1.00 C

ATOM 177 CD ARG R 530 44.849 95.563 102.612 1.00 1.00 C

ATOM 178 NE ARG R 530 45.463 95.847 103.935 1.00 1.00 N

ATOM 179 CZ ARG R 530 46.325 96.824 104.196 1.00 1.00 C

ATOM 180 NH1 ARG R 530 46.593 97.789 103.360 1.00 1.00 N

ATOM 181 NH2 ARG R 530 46.928 96.811 105.345 1.00 1.00 N

ATOM 182 C ARG R 530 42.951 91.808 100.861 1.00 1.00 C

ATOM 183 O ARG R 530 43.073 90.664 100.406 1.00 1.00 O

ATOM 184 N GLU R 531 41.891 92.571 100.624 1.00 1.00 N

ATOM 185 CA GLU R 531 40.664 92.082 99.980 1.00 1.00 C

ATOM 186 CB GLU R 531 39.605 91.787 101.082 1.00 1.00 C

ATOM 187 CG GLU R 531 39.837 90.474 101.874 1.00 1.00 C

ATOM 188 CD GLU R 531 39.172 90.365 103.234 1.00 1.00 C

ATOM 189 OE1 GLU R 531 38.269 91.152 103.590 1.00 1.00 O

ATOM 190 OE2 GLU R 531 39.581 89.474 103.997 1.00 1.00 O

ATOM 191 C GLU R 531 40.166 93.139 98.967 1.00 1.00 C

ATOM 192 O GLU R 531 40.421 94.350 99.033 1.00 1.00 O

ATOM 193 N TYR R 532 39.382 92.624 98.033 1.00 1.00 N

ATOM 194 CA TYR R 532 38.469 93.438 97.208 1.00 1.00 C

ATOM 195 CB TYR R 532 38.931 93.348 95.722 1.00 1.00 C

ATOM 196 CG TYR R 532 38.649 92.012 95.035 1.00 1.00 C

ATOM 197 CD1 TYR R 532 39.457 90.896 95.290 1.00 1.00 C

ATOM 198 CD2 TYR R 532 37.491 91.866 94.267 1.00 1.00 C

ATOM 199 CE1 TYR R 532 39.085 89.649 94.808 1.00 1.00 C

ATOM 200 CE2 TYR R 532 37.150 90.624 93.751 1.00 1.00 C

ATOM 201 CZ TYR R 532 37.939 89.511 94.035 1.00 1.00 C

ATOM 202 OH TYR R 532 37.567 88.283 93.573 1.00 1.00 O

ATOM 203 C TYR R 532 36.990 93.043 97.499 1.00 1.00 C

ATOM 204 O TYR R 532 36.684 92.022 98.132 1.00 1.00 O

ATOM 205 N VAL R 533 36.067 93.882 97.023 1.00 1.00 N

ATOM 206 CA VAL R 533 34.612 93.650 97.221 1.00 1.00 C

ATOM 207 CB VAL R 533 33.954 94.902 97.885 1.00 1.00 C

ATOM 208 CG1 VAL R 533 32.415 94.802 97.942 1.00 1.00 C

ATOM 209 CG2 VAL R 533 34.449 95.122 99.329 1.00 1.00 C

ATOM 210 C VAL R 533 34.002 93.261 95.850 1.00 1.00 C

ATOM 211 O VAL R 533 33.898 94.115 94.978 1.00 1.00 O

ATOM 212 N ASN R 534 33.525 92.019 95.676 1.00 1.00 N

ATOM 213 CA ASN R 534 32.654 91.677 94.516 1.00 1.00 C

ATOM 214 CB ASN R 534 33.179 90.427 93.788 1.00 1.00 C

ATOM 215 CG ASN R 534 32.516 90.160 92.434 1.00 1.00 C

ATOM 216 OD1 ASN R 534 31.387 89.691 92.351 1.00 1.00 O

ATOM 217 ND2 ASN R 534 33.160 90.465 91.335 1.00 1.00 N

ATOM 218 C ASN R 534 31.193 91.495 94.998 1.00 1.00 C

ATOM 219 O ASN R 534 30.916 90.572 95.769 1.00 1.00 O

ATOM 220 N ALA R 535 30.265 92.368 94.561 1.00 1.00 N

ATOM 221 CA ALA R 535 28.837 92.335 95.004 1.00 1.00 C

ATOM 222 CB ALA R 535 28.079 91.315 94.130 1.00 1.00 C

ATOM 223 C ALA R 535 28.594 92.148 96.540 1.00 1.00 C

ATOM 224 O ALA R 535 27.896 91.228 96.978 1.00 1.00 O

ATOM 225 N ARG R 536 29.250 92.994 97.359 1.00 1.00 N

ATOM 226 CA ARG R 536 29.262 92.854 98.851 1.00 1.00 C

ATOM 227 CB ARG R 536 27.853 93.083 99.462 1.00 1.00 C

ATOM 228 CG ARG R 536 27.140 94.385 99.025 1.00 1.00 C

ATOM 229 CD ARG R 536 25.766 94.491 99.688 1.00 1.00 C

ATOM 230 NE ARG R 536 24.982 95.579 99.047 1.00 1.00 N

ATOM 231 CZ ARG R 536 23.681 95.785 99.233 1.00 1.00 C

ATOM 232 NH1 ARG R 536 22.963 95.111 100.099 1.00 1.00 N

ATOM 233 NH2 ARG R 536 23.092 96.701 98.521 1.00 1.00 N

ATOM 234 C ARG R 536 29.984 91.598 99.468 1.00 1.00 C

ATOM 235 O ARG R 536 30.012 91.457 100.694 1.00 1.00 O

ATOM 236 N HIS R 537 30.610 90.718 98.668 1.00 1.00 N

ATOM 237 CA HIS R 537 31.470 89.621 99.172 1.00 1.00 C

ATOM 238 ND1 HIS R 537 29.829 86.632 99.238 1.00 1.00 N

ATOM 239 CG HIS R 537 30.092 87.620 98.298 1.00 1.00 C

ATOM 240 CB HIS R 537 31.411 88.384 98.235 1.00 1.00 C

ATOM 241 NE2 HIS R 537 27.920 87.047 97.886 1.00 1.00 N

ATOM 242 CD2 HIS R 537 28.963 87.869 97.499 1.00 1.00 C

ATOM 243 CE1 HIS R 537 28.525 86.365 98.909 1.00 1.00 C

ATOM 244 C HIS R 537 32.925 90.143 99.312 1.00 1.00 C

ATOM 245 O HIS R 537 33.522 90.636 98.348 1.00 1.00 O

ATOM 246 N CYS R 538 33.502 89.976 100.512 1.00 1.00 N

ATOM 247 CA CYS R 538 34.952 90.149 100.725 1.00 1.00 C

ATOM 248 CB CYS R 538 35.268 90.194 102.230 1.00 1.00 C

ATOM 249 SG CYS R 538 34.243 91.332 103.165 1.00 1.00 S

ATOM 250 C CYS R 538 35.713 88.947 100.110 1.00 1.00 C

ATOM 251 O CYS R 538 35.586 87.802 100.566 1.00 1.00 O

ATOM 252 N LEU R 539 36.470 89.219 99.052 1.00 1.00 N

ATOM 253 CA LEU R 539 37.247 88.173 98.352 1.00 1.00 C

ATOM 254 CB LEU R 539 36.740 88.036 96.900 1.00 1.00 C

ATOM 255 CG LEU R 539 35.279 87.549 96.728 1.00 1.00 C

ATOM 256 CD1 LEU R 539 34.863 87.688 95.263 1.00 1.00 C

ATOM 257 CD2 LEU R 539 35.054 86.101 97.192 1.00 1.00 C

ATOM 258 C LEU R 539 38.756 88.515 98.451 1.00 1.00 C

ATOM 259 O LEU R 539 39.109 89.704 98.446 1.00 1.00 O

ATOM 260 N PRO R 540 39.695 87.536 98.579 1.00 1.00 N

ATOM 261 CA PRO R 540 41.119 87.847 98.811 1.00 1.00 C

ATOM 262 CD PRO R 540 39.491 86.112 98.246 1.00 1.00 C

ATOM 263 CB PRO R 540 41.726 86.459 99.131 1.00 1.00 C

ATOM 264 CG PRO R 540 40.573 85.448 99.089 1.00 1.00 C

ATOM 265 C PRO R 540 41.743 88.535 97.566 1.00 1.00 C

ATOM 266 O PRO R 540 41.632 88.030 96.441 1.00 1.00 O

ATOM 267 N CYS R 541 42.471 89.643 97.773 1.00 1.00 N

ATOM 268 CA CYS R 541 43.522 90.046 96.810 1.00 1.00 C

ATOM 269 CB CYS R 541 44.237 91.309 97.335 1.00 1.00 C

ATOM 270 SG CYS R 541 43.182 92.756 97.168 1.00 1.00 S

ATOM 271 C CYS R 541 44.545 88.893 96.612 1.00 1.00 C

ATOM 272 O CYS R 541 44.891 88.194 97.576 1.00 1.00 O

ATOM 273 N HIS R 542 45.021 88.688 95.372 1.00 1.00 N

ATOM 274 CA HIS R 542 46.106 87.709 95.112 1.00 1.00 C

ATOM 275 ND1 HIS R 542 46.996 85.399 92.858 1.00 1.00 N

ATOM 276 CG HIS R 542 47.450 86.666 93.198 1.00 1.00 C

ATOM 277 CB HIS R 542 46.570 87.834 93.640 1.00 1.00 C

ATOM 278 NE2 HIS R 542 49.372 85.468 92.895 1.00 1.00 N

ATOM 279 CD2 HIS R 542 48.846 86.703 93.214 1.00 1.00 C

ATOM 280 CE1 HIS R 542 48.208 84.776 92.683 1.00 1.00 C

ATOM 281 C HIS R 542 47.321 87.913 96.097 1.00 1.00 C

ATOM 282 O HIS R 542 47.731 89.072 96.247 1.00 1.00 O

ATOM 283 N PRO R 543 47.910 86.886 96.777 1.00 1.00 N

ATOM 284 CA PRO R 543 49.050 87.104 97.721 1.00 1.00 C

ATOM 285 CD PRO R 543 47.492 85.475 96.665 1.00 1.00 C

ATOM 286 CB PRO R 543 49.430 85.660 98.094 1.00 1.00 C

ATOM 287 CG PRO R 543 48.172 84.820 97.862 1.00 1.00 C

ATOM 288 C PRO R 543 50.293 87.949 97.272 1.00 1.00 C

ATOM 289 O PRO R 543 51.026 88.463 98.118 1.00 1.00 O

ATOM 290 N GLU R 544 50.487 88.105 95.953 1.00 1.00 N

ATOM 291 CA GLU R 544 51.546 88.966 95.354 1.00 1.00 C

ATOM 292 CB GLU R 544 52.120 88.249 94.098 1.00 1.00 C

ATOM 293 CG GLU R 544 52.670 86.819 94.344 1.00 1.00 C

ATOM 294 CD GLU R 544 53.794 86.776 95.366 1.00 1.00 C

ATOM 295 OE1 GLU R 544 54.728 87.595 95.258 1.00 1.00 O

ATOM 296 OE2 GLU R 544 53.748 85.925 96.277 1.00 1.00 O

ATOM 297 C GLU R 544 51.165 90.448 95.039 1.00 1.00 C

ATOM 298 O GLU R 544 52.030 91.213 94.598 1.00 1.00 O

ATOM 299 N CYS R 545 49.930 90.905 95.328 1.00 1.00 N

ATOM 300 CA CYS R 545 49.636 92.345 95.475 1.00 1.00 C

ATOM 301 CB CYS R 545 48.105 92.528 95.542 1.00 1.00 C

ATOM 302 SG CYS R 545 47.276 91.875 94.079 1.00 1.00 S

ATOM 303 C CYS R 545 50.315 92.926 96.746 1.00 1.00 C

ATOM 304 O CYS R 545 50.104 92.438 97.863 1.00 1.00 O

ATOM 305 N GLN R 546 51.117 93.989 96.574 1.00 1.00 N

ATOM 306 CA GLN R 546 51.675 94.758 97.710 1.00 1.00 C

ATOM 307 CB GLN R 546 52.680 95.803 97.161 1.00 1.00 C

ATOM 308 CG GLN R 546 53.462 96.618 98.224 1.00 1.00 C

ATOM 309 CD GLN R 546 54.577 97.475 97.618 1.00 1.00 C

ATOM 310 OE1 GLN R 546 55.726 97.065 97.511 1.00 1.00 O

ATOM 311 NE2 GLN R 546 54.292 98.685 97.201 1.00 1.00 N

ATOM 312 C GLN R 546 50.513 95.431 98.509 1.00 1.00 C

ATOM 313 O GLN R 546 49.827 96.276 97.914 1.00 1.00 O

ATOM 314 N PRO R 547 50.244 95.122 99.814 1.00 1.00 N

ATOM 315 CA PRO R 547 49.230 95.857 100.602 1.00 1.00 C

ATOM 316 CD PRO R 547 50.974 94.116 100.612 1.00 1.00 C

ATOM 317 CB PRO R 547 49.258 95.153 101.967 1.00 1.00 C

ATOM 318 CG PRO R 547 50.635 94.503 102.052 1.00 1.00 C

ATOM 319 C PRO R 547 49.536 97.378 100.669 1.00 1.00 C

ATOM 320 O PRO R 547 50.670 97.803 100.913 1.00 1.00 O

ATOM 321 N GLN R 548 48.526 98.176 100.325 1.00 1.00 N

ATOM 322 CA GLN R 548 48.726 99.618 100.074 1.00 1.00 C

ATOM 323 CB GLN R 548 47.923 100.079 98.845 1.00 1.00 C

ATOM 324 CG GLN R 548 48.417 99.544 97.499 1.00 1.00 C

ATOM 325 CD GLN R 548 49.863 99.922 97.130 1.00 1.00 C

ATOM 326 OE1 GLN R 548 50.185 101.047 96.764 1.00 1.00 O

ATOM 327 NE2 GLN R 548 50.768 98.979 97.175 1.00 1.00 N

ATOM 328 C GLN R 548 48.347 100.470 101.292 1.00 1.00 C

ATOM 329 O GLN R 548 47.430 100.167 102.063 1.00 1.00 O

ATOM 330 N ASN R 549 49.063 101.583 101.420 1.00 1.00 N

ATOM 331 CA ASN R 549 48.849 102.531 102.527 1.00 1.00 C

ATOM 332 CB ASN R 549 50.229 102.929 103.088 1.00 1.00 C

ATOM 333 CG ASN R 549 50.777 101.933 104.120 1.00 1.00 C

ATOM 334 OD1 ASN R 549 50.169 100.942 104.519 1.00 1.00 O

ATOM 335 ND2 ASN R 549 51.946 102.204 104.625 1.00 1.00 N

ATOM 336 C ASN R 549 48.009 103.724 102.035 1.00 1.00 C

ATOM 337 O ASN R 549 48.415 104.495 101.159 1.00 1.00 O

ATOM 338 N GLY R 550 46.796 103.813 102.593 1.00 1.00 N

ATOM 339 CA GLY R 550 45.795 104.792 102.124 1.00 1.00 C

ATOM 340 C GLY R 550 45.112 104.586 100.760 1.00 1.00 C

ATOM 341 O GLY R 550 44.300 105.403 100.345 1.00 1.00 O

ATOM 342 N SER R 551 45.423 103.498 100.074 1.00 1.00 N

ATOM 343 CA SER R 551 44.822 103.151 98.790 1.00 1.00 C

ATOM 344 CB SER R 551 45.931 103.304 97.737 1.00 1.00 C

ATOM 345 OG SER R 551 45.370 103.177 96.444 1.00 1.00 O

ATOM 346 C SER R 551 44.213 101.737 98.842 1.00 1.00 C

ATOM 347 O SER R 551 44.551 100.894 99.685 1.00 1.00 O

ATOM 348 N VAL R 552 43.353 101.451 97.861 1.00 1.00 N

ATOM 349 CA VAL R 552 42.972 100.053 97.532 1.00 1.00 C

ATOM 350 CB VAL R 552 41.813 100.127 96.471 1.00 1.00 C

ATOM 351 CG1 VAL R 552 41.723 98.967 95.455 1.00 1.00 C

ATOM 352 CG2 VAL R 552 40.462 100.200 97.200 1.00 1.00 C

ATOM 353 C VAL R 552 44.221 99.177 97.159 1.00 1.00 C

ATOM 354 O VAL R 552 45.202 99.657 96.588 1.00 1.00 O

ATOM 355 N THR R 553 44.182 97.891 97.527 1.00 1.00 N

ATOM 356 CA THR R 553 45.264 96.917 97.181 1.00 1.00 C

ATOM 357 CB THR R 553 45.509 95.981 98.395 1.00 1.00 C

ATOM 358 OG1 THR R 553 46.086 96.754 99.437 1.00 1.00 O

ATOM 359 CG2 THR R 553 46.489 94.824 98.127 1.00 1.00 C

ATOM 360 C THR R 553 45.079 96.160 95.832 1.00 1.00 C

ATOM 361 O THR R 553 46.081 95.883 95.160 1.00 1.00 O

ATOM 362 N CYS R 554 43.857 95.782 95.449 1.00 1.00 N

ATOM 363 CA CYS R 554 43.610 95.158 94.133 1.00 1.00 C

ATOM 364 CB CYS R 554 43.833 93.635 94.254 1.00 1.00 C

ATOM 365 SG CYS R 554 42.564 92.858 95.271 1.00 1.00 S

ATOM 366 C CYS R 554 42.212 95.512 93.579 1.00 1.00 C

ATOM 367 O CYS R 554 41.297 95.888 94.307 1.00 1.00 O

ATOM 368 N PHE R 555 42.073 95.339 92.267 1.00 1.00 N

ATOM 369 CA PHE R 555 40.782 95.512 91.521 1.00 1.00 C

ATOM 370 CB PHE R 555 41.074 96.152 90.124 1.00 1.00 C

ATOM 371 CG PHE R 555 42.145 97.265 90.069 1.00 1.00 C

ATOM 372 CD1 PHE R 555 41.935 98.482 90.721 1.00 1.00 C

ATOM 373 CD2 PHE R 555 43.416 96.966 89.566 1.00 1.00 C

ATOM 374 CE1 PHE R 555 42.978 99.392 90.853 1.00 1.00 C

ATOM 375 CE2 PHE R 555 44.463 97.870 89.719 1.00 1.00 C

ATOM 376 CZ PHE R 555 44.241 99.087 90.358 1.00 1.00 C

ATOM 377 C PHE R 555 39.936 94.199 91.348 1.00 1.00 C

ATOM 378 O PHE R 555 38.765 94.254 90.962 1.00 1.00 O

ATOM 379 N GLY R 556 40.554 93.040 91.602 1.00 1.00 N

ATOM 380 CA GLY R 556 39.938 91.715 91.482 1.00 1.00 C

ATOM 381 C GLY R 556 40.865 90.583 92.014 1.00 1.00 C

ATOM 382 O GLY R 556 41.848 90.861 92.717 1.00 1.00 O

ATOM 383 N PRO R 557 40.570 89.284 91.748 1.00 1.00 N

ATOM 384 CA PRO R 557 41.330 88.154 92.353 1.00 1.00 C

ATOM 385 CD PRO R 557 39.443 88.850 90.895 1.00 1.00 C

ATOM 386 CB PRO R 557 40.330 86.996 92.177 1.00 1.00 C

ATOM 387 CG PRO R 557 39.556 87.328 90.896 1.00 1.00 C

ATOM 388 C PRO R 557 42.729 87.816 91.761 1.00 1.00 C

ATOM 389 O PRO R 557 43.528 87.195 92.463 1.00 1.00 O

ATOM 390 N GLU R 558 43.002 88.154 90.490 1.00 1.00 N

ATOM 391 CA GLU R 558 44.205 87.689 89.762 1.00 1.00 C

ATOM 392 CB GLU R 558 43.860 87.634 88.243 1.00 1.00 C

ATOM 393 CG GLU R 558 42.850 86.519 87.853 1.00 1.00 C

ATOM 394 CD GLU R 558 42.460 86.469 86.386 1.00 1.00 C

ATOM 395 OE1 GLU R 558 41.512 87.182 85.993 1.00 1.00 O

ATOM 396 OE2 GLU R 558 43.060 85.671 85.636 1.00 1.00 O

ATOM 397 C GLU R 558 45.472 88.548 90.069 1.00 1.00 C

ATOM 398 O GLU R 558 45.425 89.730 90.430 1.00 1.00 O

ATOM 399 N ALA R 559 46.644 87.922 89.891 1.00 1.00 N

ATOM 400 CA ALA R 559 47.962 88.598 90.043 1.00 1.00 C

ATOM 401 CB ALA R 559 48.995 87.468 89.918 1.00 1.00 C

ATOM 402 C ALA R 559 48.322 89.785 89.085 1.00 1.00 C

ATOM 403 O ALA R 559 49.242 90.553 89.377 1.00 1.00 O

ATOM 404 N ASP R 560 47.605 89.933 87.963 1.00 1.00 N

ATOM 405 CA ASP R 560 47.595 91.164 87.125 1.00 1.00 C

ATOM 406 CB ASP R 560 47.489 90.689 85.654 1.00 1.00 C

ATOM 407 CG ASP R 560 46.197 90.032 85.157 1.00 1.00 C

ATOM 408 OD1 ASP R 560 45.349 89.624 85.980 1.00 1.00 O

ATOM 409 OD2 ASP R 560 46.046 89.913 83.923 1.00 1.00 O

ATOM 410 C ASP R 560 46.525 92.260 87.484 1.00 1.00 C

ATOM 411 O ASP R 560 46.448 93.297 86.819 1.00 1.00 O

ATOM 412 N GLN R 561 45.740 92.061 88.554 1.00 1.00 N

ATOM 413 CA GLN R 561 44.836 93.090 89.141 1.00 1.00 C

ATOM 414 CB GLN R 561 43.467 92.390 89.331 1.00 1.00 C

ATOM 415 CG GLN R 561 42.701 92.167 88.004 1.00 1.00 C

ATOM 416 CD GLN R 561 41.479 91.264 88.146 1.00 1.00 C

ATOM 417 OE1 GLN R 561 41.517 90.186 88.727 1.00 1.00 O

ATOM 418 NE2 GLN R 561 40.351 91.656 87.611 1.00 1.00 N

ATOM 419 C GLN R 561 45.355 93.736 90.467 1.00 1.00 C

ATOM 420 O GLN R 561 44.601 94.436 91.150 1.00 1.00 O

ATOM 421 N CYS R 562 46.649 93.579 90.792 1.00 1.00 N

ATOM 422 CA CYS R 562 47.331 94.364 91.826 1.00 1.00 C

ATOM 423 CB CYS R 562 48.700 93.693 92.051 1.00 1.00 C

ATOM 424 SG CYS R 562 48.527 91.966 92.528 1.00 1.00 S

ATOM 425 C CYS R 562 47.565 95.821 91.364 1.00 1.00 C

ATOM 426 O CYS R 562 47.842 96.114 90.195 1.00 1.00 O

ATOM 427 N VAL R 563 47.574 96.726 92.333 1.00 1.00 N

ATOM 428 CA VAL R 563 48.121 98.097 92.109 1.00 1.00 C

ATOM 429 CB VAL R 563 47.586 99.134 93.153 1.00 1.00 C

ATOM 430 CG1 VAL R 563 46.261 98.766 93.870 1.00 1.00 C

ATOM 431 CG2 VAL R 563 48.636 99.511 94.221 1.00 1.00 C

ATOM 432 C VAL R 563 49.692 98.122 91.918 1.00 1.00 C

ATOM 433 O VAL R 563 50.258 98.942 91.190 1.00 1.00 O

ATOM 434 N ALA R 564 50.378 97.320 92.741 1.00 1.00 N

ATOM 435 CA ALA R 564 51.839 97.224 92.800 1.00 1.00 C

ATOM 436 CB ALA R 564 52.377 98.283 93.783 1.00 1.00 C

ATOM 437 C ALA R 564 52.184 95.777 93.231 1.00 1.00 C

ATOM 438 O ALA R 564 51.496 95.140 94.041 1.00 1.00 O

ATOM 439 N CYS R 565 53.277 95.260 92.671 1.00 1.00 N

ATOM 440 CA CYS R 565 53.786 93.927 93.042 1.00 1.00 C

ATOM 441 CB CYS R 565 54.723 93.486 91.905 1.00 1.00 C

ATOM 442 SG CYS R 565 53.789 93.256 90.380 1.00 1.00 S

ATOM 443 C CYS R 565 54.491 93.946 94.423 1.00 1.00 C

ATOM 444 O CYS R 565 55.343 94.798 94.696 1.00 1.00 O

ATOM 445 N ALA R 566 54.135 92.985 95.287 1.00 1.00 N

ATOM 446 CA ALA R 566 54.900 92.714 96.533 1.00 1.00 C

ATOM 447 CB ALA R 566 54.126 91.605 97.272 1.00 1.00 C

ATOM 448 C ALA R 566 56.406 92.344 96.340 1.00 1.00 C

ATOM 449 O ALA R 566 57.232 92.690 97.187 1.00 1.00 O

ATOM 450 N HIS R 567 56.744 91.642 95.242 1.00 1.00 N

ATOM 451 CA HIS R 567 58.113 91.138 94.984 1.00 1.00 C

ATOM 452 ND1 HIS R 567 58.963 89.372 97.743 1.00 1.00 N

ATOM 453 CG HIS R 567 57.961 89.265 96.791 1.00 1.00 C

ATOM 454 CB HIS R 567 58.153 89.613 95.318 1.00 1.00 C

ATOM 455 NE2 HIS R 567 56.882 88.815 98.753 1.00 1.00 N

ATOM 456 CD2 HIS R 567 56.731 88.943 97.390 1.00 1.00 C

ATOM 457 CE1 HIS R 567 58.219 89.093 98.862 1.00 1.00 C

ATOM 458 C HIS R 567 58.528 91.534 93.538 1.00 1.00 C

ATOM 459 O HIS R 567 59.096 92.610 93.329 1.00 1.00 O

ATOM 460 N TYR R 568 58.244 90.675 92.549 1.00 1.00 N

ATOM 461 CA TYR R 568 58.635 90.891 91.136 1.00 1.00 C

ATOM 462 CB TYR R 568 59.680 89.805 90.718 1.00 1.00 C

ATOM 463 CG TYR R 568 61.002 89.823 91.510 1.00 1.00 C

ATOM 464 CD1 TYR R 568 61.950 90.823 91.288 1.00 1.00 C

ATOM 465 CD2 TYR R 568 61.214 88.898 92.536 1.00 1.00 C

ATOM 466 CE1 TYR R 568 63.080 90.920 92.096 1.00 1.00 C

ATOM 467 CE2 TYR R 568 62.351 88.982 93.335 1.00 1.00 C

ATOM 468 CZ TYR R 568 63.283 89.993 93.115 1.00 1.00 C

ATOM 469 OH TYR R 568 64.393 90.083 93.910 1.00 1.00 O

ATOM 470 C TYR R 568 57.360 90.878 90.248 1.00 1.00 C

ATOM 471 O TYR R 568 56.263 90.470 90.650 1.00 1.00 O

ATOM 472 N LYS R 569 57.512 91.337 89.005 1.00 1.00 N

ATOM 473 CA LYS R 569 56.433 91.256 87.995 1.00 1.00 C

ATOM 474 CB LYS R 569 55.984 92.697 87.649 1.00 1.00 C

ATOM 475 CG LYS R 569 54.663 92.738 86.844 1.00 1.00 C

ATOM 476 CD LYS R 569 54.263 94.140 86.353 1.00 1.00 C

ATOM 477 CE LYS R 569 53.709 95.050 87.465 1.00 1.00 C

ATOM 478 NZ LYS R 569 53.418 96.391 86.897 1.00 1.00 N

ATOM 479 C LYS R 569 56.942 90.446 86.782 1.00 1.00 C

ATOM 480 O LYS R 569 57.896 90.850 86.124 1.00 1.00 O

ATOM 481 N ASP R 570 56.295 89.333 86.436 1.00 1.00 N

ATOM 482 CA ASP R 570 56.428 88.752 85.081 1.00 1.00 C

ATOM 483 CB ASP R 570 56.568 87.214 85.187 1.00 1.00 C

ATOM 484 CG ASP R 570 56.972 86.562 83.868 1.00 1.00 C

ATOM 485 OD1 ASP R 570 56.976 87.235 82.816 1.00 1.00 O

ATOM 486 OD2 ASP R 570 57.332 85.368 83.849 1.00 1.00 O

ATOM 487 C ASP R 570 55.210 89.279 84.247 1.00 1.00 C

ATOM 488 O ASP R 570 54.127 88.696 84.384 1.00 1.00 O

ATOM 489 N PRO R 571 55.291 90.401 83.459 1.00 1.00 N

ATOM 490 CA PRO R 571 54.092 91.120 82.966 1.00 1.00 C

ATOM 491 CD PRO R 571 56.544 91.082 83.079 1.00 1.00 C

ATOM 492 CB PRO R 571 54.669 92.328 82.209 1.00 1.00 C

ATOM 493 CG PRO R 571 56.087 92.502 82.750 1.00 1.00 C

ATOM 494 C PRO R 571 53.093 90.280 82.098 1.00 1.00 C

ATOM 495 O PRO R 571 53.549 89.545 81.214 1.00 1.00 O

ATOM 496 N PRO R 572 51.752 90.327 82.325 1.00 1.00 N

ATOM 497 CA PRO R 572 51.086 91.299 83.249 1.00 1.00 C

ATOM 498 CD PRO R 572 50.813 89.452 81.597 1.00 1.00 C

ATOM 499 CB PRO R 572 49.695 91.331 82.589 1.00 1.00 C

ATOM 500 CG PRO R 572 49.448 89.896 82.117 1.00 1.00 C

ATOM 501 C PRO R 572 51.054 90.986 84.790 1.00 1.00 C

ATOM 502 O PRO R 572 50.515 91.784 85.559 1.00 1.00 O

ATOM 503 N PHE R 573 51.646 89.873 85.249 1.00 1.00 N

ATOM 504 CA PHE R 573 51.396 89.293 86.590 1.00 1.00 C

ATOM 505 CB PHE R 573 51.354 87.737 86.470 1.00 1.00 C

ATOM 506 CG PHE R 573 50.242 87.160 85.575 1.00 1.00 C

ATOM 507 CD1 PHE R 573 48.904 87.277 85.957 1.00 1.00 C

ATOM 508 CD2 PHE R 573 50.552 86.588 84.338 1.00 1.00 C

ATOM 509 CE1 PHE R 573 47.886 86.853 85.111 1.00 1.00 C

ATOM 510 CE2 PHE R 573 49.533 86.152 83.492 1.00 1.00 C

ATOM 511 CZ PHE R 573 48.201 86.291 83.878 1.00 1.00 C

ATOM 512 C PHE R 573 52.482 89.689 87.622 1.00 1.00 C

ATOM 513 O PHE R 573 53.681 89.511 87.392 1.00 1.00 O

ATOM 514 N CYS R 574 52.060 90.107 88.823 1.00 1.00 N

ATOM 515 CA CYS R 574 52.915 90.025 90.031 1.00 1.00 C

ATOM 516 CB CYS R 574 52.174 90.748 91.164 1.00 1.00 C

ATOM 517 SG CYS R 574 51.983 92.497 90.791 1.00 1.00 S

ATOM 518 C CYS R 574 53.231 88.552 90.433 1.00 1.00 C

ATOM 519 O CYS R 574 52.335 87.709 90.533 1.00 1.00 O

ATOM 520 N VAL R 575 54.513 88.241 90.628 1.00 1.00 N

ATOM 521 CA VAL R 575 54.966 86.866 90.974 1.00 1.00 C

ATOM 522 CB VAL R 575 55.540 86.065 89.749 1.00 1.00 C

ATOM 523 CG1 VAL R 575 54.462 85.715 88.700 1.00 1.00 C

ATOM 524 CG2 VAL R 575 56.750 86.715 89.042 1.00 1.00 C

ATOM 525 C VAL R 575 55.961 86.926 92.168 1.00 1.00 C

ATOM 526 O VAL R 575 56.779 87.848 92.298 1.00 1.00 O

ATOM 527 N ALA R 576 55.943 85.863 92.991 1.00 1.00 N

ATOM 528 CA ALA R 576 56.944 85.669 94.072 1.00 1.00 C

ATOM 529 CB ALA R 576 56.531 84.405 94.858 1.00 1.00 C

ATOM 530 C ALA R 576 58.411 85.595 93.561 1.00 1.00 C

ATOM 531 O ALA R 576 59.261 86.379 93.989 1.00 1.00 O

ATOM 532 N ARG R 577 58.680 84.669 92.629 1.00 1.00 N

ATOM 533 CA ARG R 577 60.011 84.505 92.008 1.00 1.00 C

ATOM 534 CB ARG R 577 60.538 83.072 92.308 1.00 1.00 C

ATOM 535 CG ARG R 577 60.625 82.704 93.811 1.00 1.00 C

ATOM 536 CD ARG R 577 61.457 81.443 94.069 1.00 1.00 C

ATOM 537 NE ARG R 577 61.340 81.075 95.506 1.00 1.00 N

ATOM 538 CZ ARG R 577 62.137 80.225 96.152 1.00 1.00 C

ATOM 539 NH1 ARG R 577 63.166 79.635 95.596 1.00 1.00 N

ATOM 540 NH2 ARG R 577 61.880 79.969 97.401 1.00 1.00 N

ATOM 541 C ARG R 577 59.929 84.770 90.479 1.00 1.00 C

ATOM 542 O ARG R 577 58.927 84.472 89.815 1.00 1.00 O

ATOM 543 N CYS R 578 61.057 85.207 89.894 1.00 1.00 N

ATOM 544 CA CYS R 578 61.307 85.000 88.447 1.00 1.00 C

ATOM 545 CB CYS R 578 62.645 85.661 88.067 1.00 1.00 C

ATOM 546 SG CYS R 578 62.452 87.465 87.919 1.00 1.00 S

ATOM 547 C CYS R 578 61.304 83.476 88.049 1.00 1.00 C

ATOM 548 O CYS R 578 62.008 82.701 88.714 1.00 1.00 O

ATOM 549 N PRO R 579 60.552 83.001 87.008 1.00 1.00 N

ATOM 550 CA PRO R 579 60.580 81.579 86.581 1.00 1.00 C

ATOM 551 CD PRO R 579 59.539 83.796 86.285 1.00 1.00 C

ATOM 552 CB PRO R 579 59.650 81.556 85.356 1.00 1.00 C

ATOM 553 CG PRO R 579 58.714 82.746 85.545 1.00 1.00 C

ATOM 554 C PRO R 579 61.987 81.031 86.226 1.00 1.00 C

ATOM 555 O PRO R 579 62.713 81.595 85.405 1.00 1.00 O

ATOM 556 N SER R 580 62.377 79.946 86.886 1.00 1.00 N

ATOM 557 CA SER R 580 63.666 79.270 86.613 1.00 1.00 C

ATOM 558 CB SER R 580 64.811 80.032 87.355 1.00 1.00 C

ATOM 559 OG SER R 580 64.569 80.131 88.759 1.00 1.00 O

ATOM 560 C SER R 580 63.454 77.752 86.875 1.00 1.00 C

ATOM 561 O SER R 580 62.484 77.160 86.385 1.00 1.00 O

ATOM 562 N GLY R 581 64.348 77.099 87.618 1.00 1.00 N

ATOM 563 CA GLY R 581 64.283 75.639 87.807 1.00 1.00 C

ATOM 564 C GLY R 581 65.527 75.052 88.484 1.00 1.00 C

ATOM 565 O GLY R 581 66.284 75.711 89.204 1.00 1.00 O

ATOM 566 N VAL R 582 65.708 73.762 88.219 1.00 1.00 N

ATOM 567 CA VAL R 582 66.902 72.998 88.667 1.00 1.00 C

ATOM 568 CB VAL R 582 66.433 71.526 88.934 1.00 1.00 C

ATOM 569 CG1 VAL R 582 66.072 70.703 87.671 1.00 1.00 C

ATOM 570 CG2 VAL R 582 67.442 70.724 89.780 1.00 1.00 C

ATOM 571 C VAL R 582 68.088 73.154 87.660 1.00 1.00 C

ATOM 572 O VAL R 582 67.885 73.456 86.483 1.00 1.00 O

ATOM 573 N LYS R 583 69.327 72.913 88.120 1.00 1.00 N

ATOM 574 CA LYS R 583 70.550 72.954 87.266 1.00 1.00 C

ATOM 575 CB LYS R 583 71.751 72.326 88.030 1.00 1.00 C

ATOM 576 CG LYS R 583 71.579 70.882 88.586 1.00 1.00 C

ATOM 577 CD LYS R 583 72.841 69.995 88.474 1.00 1.00 C

ATOM 578 CE LYS R 583 72.874 69.048 87.257 1.00 1.00 C

ATOM 579 NZ LYS R 583 72.979 69.736 85.945 1.00 1.00 N

ATOM 580 C LYS R 583 70.419 72.320 85.832 1.00 1.00 C

ATOM 581 O LYS R 583 70.304 71.090 85.762 1.00 1.00 O

ATOM 582 N PRO R 584 70.439 73.073 84.689 1.00 1.00 N

ATOM 583 CA PRO R 584 70.262 72.479 83.344 1.00 1.00 C

ATOM 584 CD PRO R 584 70.412 74.549 84.657 1.00 1.00 C

ATOM 585 CB PRO R 584 70.349 73.686 82.396 1.00 1.00 C

ATOM 586 CG PRO R 584 69.895 74.863 83.253 1.00 1.00 C

ATOM 587 C PRO R 584 71.280 71.362 82.997 1.00 1.00 C

ATOM 588 O PRO R 584 72.501 71.536 83.073 1.00 1.00 O

ATOM 589 N ASP R 585 70.727 70.196 82.676 1.00 1.00 N

ATOM 590 CA ASP R 585 71.499 69.077 82.086 1.00 1.00 C

ATOM 591 CB ASP R 585 70.981 67.773 82.764 1.00 1.00 C

ATOM 592 CG ASP R 585 71.466 67.614 84.200 1.00 1.00 C

ATOM 593 OD1 ASP R 585 70.646 67.619 85.139 1.00 1.00 O

ATOM 594 OD2 ASP R 585 72.700 67.586 84.407 1.00 1.00 O

ATOM 595 C ASP R 585 71.378 69.133 80.530 1.00 1.00 C

ATOM 596 O ASP R 585 70.834 70.086 79.950 1.00 1.00 O

ATOM 597 N LEU R 586 71.870 68.097 79.827 1.00 1.00 N

ATOM 598 CA LEU R 586 71.641 67.966 78.361 1.00 1.00 C

ATOM 599 CB LEU R 586 72.460 66.826 77.723 1.00 1.00 C

ATOM 600 CG LEU R 586 74.002 66.935 77.727 1.00 1.00 C

ATOM 601 CD1 LEU R 586 74.556 65.689 77.017 1.00 1.00 C

ATOM 602 CD2 LEU R 586 74.533 68.205 77.032 1.00 1.00 C

ATOM 603 C LEU R 586 70.128 67.767 78.036 1.00 1.00 C

ATOM 604 O LEU R 586 69.535 66.695 78.185 1.00 1.00 O

ATOM 605 N SER R 587 69.531 68.889 77.652 1.00 1.00 N

ATOM 606 CA SER R 587 68.078 69.073 77.484 1.00 1.00 C

ATOM 607 CB SER R 587 67.316 68.874 78.831 1.00 1.00 C

ATOM 608 OG SER R 587 67.727 69.823 79.818 1.00 1.00 O

ATOM 609 C SER R 587 67.879 70.495 76.869 1.00 1.00 C

ATOM 610 O SER R 587 68.807 71.301 76.700 1.00 1.00 O

ATOM 611 N TYR R 588 66.629 70.807 76.536 1.00 1.00 N

ATOM 612 CA TYR R 588 66.238 72.178 76.149 1.00 1.00 C

ATOM 613 CB TYR R 588 65.925 72.167 74.632 1.00 1.00 C

ATOM 614 CG TYR R 588 65.889 73.571 74.028 1.00 1.00 C

ATOM 615 CD1 TYR R 588 67.075 74.177 73.600 1.00 1.00 C

ATOM 616 CD2 TYR R 588 64.687 74.280 73.954 1.00 1.00 C

ATOM 617 CE1 TYR R 588 67.057 75.478 73.109 1.00 1.00 C

ATOM 618 CE2 TYR R 588 64.674 75.581 73.457 1.00 1.00 C

ATOM 619 CZ TYR R 588 65.857 76.182 73.045 1.00 1.00 C

ATOM 620 OH TYR R 588 65.835 77.483 72.623 1.00 1.00 O

ATOM 621 C TYR R 588 65.044 72.578 77.052 1.00 1.00 C

ATOM 622 O TYR R 588 63.983 71.945 77.012 1.00 1.00 O

ATOM 623 N MET R 589 65.227 73.615 77.877 1.00 1.00 N

ATOM 624 CA MET R 589 64.153 74.115 78.771 1.00 1.00 C

ATOM 625 CB MET R 589 64.525 73.846 80.253 1.00 1.00 C

ATOM 626 CG MET R 589 64.388 72.367 80.670 1.00 1.00 C

ATOM 627 SD MET R 589 64.723 72.177 82.430 1.00 1.00 S

ATOM 628 CE MET R 589 66.514 71.997 82.407 1.00 1.00 C

ATOM 629 C MET R 589 63.914 75.609 78.398 1.00 1.00 C

ATOM 630 O MET R 589 64.709 76.441 78.826 1.00 1.00 O

ATOM 631 N PRO R 590 62.884 76.018 77.601 1.00 1.00 N

ATOM 632 CA PRO R 590 62.731 77.439 77.175 1.00 1.00 C

ATOM 633 CD PRO R 590 61.992 75.088 76.882 1.00 1.00 C

ATOM 634 CB PRO R 590 61.547 77.371 76.190 1.00 1.00 C

ATOM 635 CG PRO R 590 61.466 75.920 75.717 1.00 1.00 C

ATOM 636 C PRO R 590 62.530 78.554 78.252 1.00 1.00 C

ATOM 637 O PRO R 590 62.918 79.698 78.001 1.00 1.00 O

ATOM 638 N ILE R 591 61.891 78.265 79.406 1.00 1.00 N

ATOM 639 CA ILE R 591 61.457 79.323 80.370 1.00 1.00 C

ATOM 640 CB ILE R 591 59.966 79.197 80.883 1.00 1.00 C

ATOM 641 CG2 ILE R 591 59.426 80.608 81.237 1.00 1.00 C

ATOM 642 CG1 ILE R 591 58.931 78.489 79.950 1.00 1.00 C

ATOM 643 CD1 ILE R 591 58.797 76.979 80.226 1.00 1.00 C

ATOM 644 C ILE R 591 62.501 79.477 81.530 1.00 1.00 C

ATOM 645 O ILE R 591 62.341 78.931 82.626 1.00 1.00 O

ATOM 646 N TRP R 592 63.546 80.282 81.279 1.00 1.00 N

ATOM 647 CA TRP R 592 64.470 80.788 82.335 1.00 1.00 C

ATOM 648 CB TRP R 592 65.961 80.396 82.133 1.00 1.00 C

ATOM 649 CG TRP R 592 66.231 78.903 82.125 1.00 1.00 C

ATOM 650 CD2 TRP R 592 66.452 78.043 83.178 1.00 1.00 C

ATOM 651 CD1 TRP R 592 66.037 78.094 81.002 1.00 1.00 C

ATOM 652 NE1 TRP R 592 66.064 76.737 81.334 1.00 1.00 N

ATOM 653 CE2 TRP R 592 66.280 76.725 82.691 1.00 1.00 C

ATOM 654 CE3 TRP R 592 66.722 78.274 84.549 1.00 1.00 C

ATOM 655 CZ2 TRP R 592 66.258 75.639 83.583 1.00 1.00 C

ATOM 656 CZ3 TRP R 592 66.790 77.171 85.401 1.00 1.00 C

ATOM 657 CH2 TRP R 592 66.514 75.881 84.931 1.00 1.00 C

ATOM 658 C TRP R 592 64.379 82.327 82.284 1.00 1.00 C

ATOM 659 O TRP R 592 64.631 82.950 81.246 1.00 1.00 O

ATOM 660 N LYS R 593 64.023 82.940 83.408 1.00 1.00 N

ATOM 661 CA LYS R 593 63.898 84.407 83.520 1.00 1.00 C

ATOM 662 CB LYS R 593 62.391 84.756 83.578 1.00 1.00 C

ATOM 663 CG LYS R 593 61.710 84.782 82.193 1.00 1.00 C

ATOM 664 CD LYS R 593 60.176 84.749 82.251 1.00 1.00 C

ATOM 665 CE LYS R 593 59.546 85.652 81.180 1.00 1.00 C

ATOM 666 NZ LYS R 593 58.086 85.418 81.184 1.00 1.00 N

ATOM 667 C LYS R 593 64.686 84.924 84.748 1.00 1.00 C

ATOM 668 O LYS R 593 64.891 84.236 85.756 1.00 1.00 O

ATOM 669 N PHE R 594 65.122 86.181 84.637 1.00 1.00 N

ATOM 670 CA PHE R 594 65.888 86.870 85.699 1.00 1.00 C

ATOM 671 CB PHE R 594 67.382 86.975 85.278 1.00 1.00 C

ATOM 672 CG PHE R 594 67.764 88.037 84.228 1.00 1.00 C

ATOM 673 CD1 PHE R 594 67.646 87.773 82.860 1.00 1.00 C

ATOM 674 CD2 PHE R 594 68.203 89.297 84.648 1.00 1.00 C

ATOM 675 CE1 PHE R 594 67.977 88.752 81.926 1.00 1.00 C

ATOM 676 CE2 PHE R 594 68.518 90.279 83.714 1.00 1.00 C

ATOM 677 CZ PHE R 594 68.411 90.004 82.353 1.00 1.00 C

ATOM 678 C PHE R 594 65.221 88.231 86.091 1.00 1.00 C

ATOM 679 O PHE R 594 64.636 88.885 85.216 1.00 1.00 O

ATOM 680 N PRO R 595 65.326 88.739 87.355 1.00 1.00 N

ATOM 681 CA PRO R 595 64.902 90.119 87.691 1.00 1.00 C

ATOM 682 CD PRO R 595 65.948 88.023 88.487 1.00 1.00 C

ATOM 683 CB PRO R 595 65.277 90.242 89.186 1.00 1.00 C

ATOM 684 CG PRO R 595 65.533 88.830 89.712 1.00 1.00 C

ATOM 685 C PRO R 595 65.591 91.233 86.844 1.00 1.00 C

ATOM 686 O PRO R 595 66.820 91.361 86.855 1.00 1.00 O

ATOM 687 N ASP R 596 64.797 92.093 86.189 1.00 1.00 N

ATOM 688 CA ASP R 596 65.259 93.457 85.820 1.00 1.00 C

ATOM 689 CB ASP R 596 64.143 94.140 84.981 1.00 1.00 C

ATOM 690 CG ASP R 596 64.531 95.426 84.268 1.00 1.00 C

ATOM 691 OD1 ASP R 596 65.580 95.446 83.592 1.00 1.00 O

ATOM 692 OD2 ASP R 596 63.816 96.433 84.429 1.00 1.00 O

ATOM 693 C ASP R 596 65.632 94.300 87.088 1.00 1.00 C

ATOM 694 O ASP R 596 65.277 93.976 88.231 1.00 1.00 O

ATOM 695 N GLU R 597 66.309 95.427 86.861 1.00 1.00 N

ATOM 696 CA GLU R 597 66.504 96.447 87.926 1.00 1.00 C

ATOM 697 CB GLU R 597 67.580 97.457 87.447 1.00 1.00 C

ATOM 698 CG GLU R 597 68.608 97.846 88.546 1.00 1.00 C

ATOM 699 CD GLU R 597 68.571 99.285 89.032 1.00 1.00 C

ATOM 700 OE1 GLU R 597 68.657 100.211 88.199 1.00 1.00 O

ATOM 701 OE2 GLU R 597 68.507 99.492 90.262 1.00 1.00 O

ATOM 702 C GLU R 597 65.199 97.133 88.467 1.00 1.00 C

ATOM 703 O GLU R 597 65.094 97.371 89.673 1.00 1.00 O

ATOM 704 N GLU R 598 64.169 97.350 87.620 1.00 1.00 N

ATOM 705 CA GLU R 598 62.790 97.698 88.087 1.00 1.00 C

ATOM 706 CB GLU R 598 61.992 98.211 86.853 1.00 1.00 C

ATOM 707 CG GLU R 598 62.498 99.533 86.216 1.00 1.00 C

ATOM 708 CD GLU R 598 61.649 100.141 85.103 1.00 1.00 C

ATOM 709 OE1 GLU R 598 60.581 99.599 84.739 1.00 1.00 O

ATOM 710 OE2 GLU R 598 62.060 101.204 84.594 1.00 1.00 O

ATOM 711 C GLU R 598 61.973 96.567 88.822 1.00 1.00 C

ATOM 712 O GLU R 598 60.845 96.808 89.266 1.00 1.00 O

ATOM 713 N GLY R 599 62.509 95.339 88.940 1.00 1.00 N

ATOM 714 CA GLY R 599 61.756 94.159 89.426 1.00 1.00 C

ATOM 715 C GLY R 599 60.938 93.337 88.398 1.00 1.00 C

ATOM 716 O GLY R 599 60.192 92.449 88.814 1.00 1.00 O

ATOM 717 N ALA R 600 61.055 93.592 87.085 1.00 1.00 N

ATOM 718 CA ALA R 600 60.296 92.857 86.051 1.00 1.00 C

ATOM 719 CB ALA R 600 59.788 93.861 85.006 1.00 1.00 C

ATOM 720 C ALA R 600 61.109 91.693 85.419 1.00 1.00 C

ATOM 721 O ALA R 600 62.242 91.874 84.981 1.00 1.00 O

ATOM 722 N CYS R 601 60.534 90.489 85.333 1.00 1.00 N

ATOM 723 CA CYS R 601 61.231 89.295 84.797 1.00 1.00 C

ATOM 724 CB CYS R 601 60.310 88.080 85.025 1.00 1.00 C

ATOM 725 SG CYS R 601 59.871 87.799 86.778 1.00 1.00 S

ATOM 726 C CYS R 601 61.601 89.429 83.284 1.00 1.00 C

ATOM 727 O CYS R 601 60.730 89.403 82.406 1.00 1.00 O

ATOM 728 N GLN R 602 62.903 89.566 82.991 1.00 1.00 N

ATOM 729 CA GLN R 602 63.413 89.489 81.598 1.00 1.00 C

ATOM 730 CB GLN R 602 64.625 90.434 81.368 1.00 1.00 C

ATOM 731 CG GLN R 602 64.446 91.932 81.718 1.00 1.00 C

ATOM 732 CD GLN R 602 63.188 92.615 81.160 1.00 1.00 C

ATOM 733 OE1 GLN R 602 63.022 92.794 79.958 1.00 1.00 O

ATOM 734 NE2 GLN R 602 62.251 92.984 82.001 1.00 1.00 N

ATOM 735 C GLN R 602 63.799 88.013 81.269 1.00 1.00 C

ATOM 736 O GLN R 602 64.295 87.320 82.168 1.00 1.00 O

ATOM 737 N PRO R 603 63.644 87.460 80.032 1.00 1.00 N

ATOM 738 CA PRO R 603 64.195 86.124 79.693 1.00 1.00 C

ATOM 739 CD PRO R 603 63.125 88.162 78.844 1.00 1.00 C

ATOM 740 CB PRO R 603 63.633 85.860 78.281 1.00 1.00 C

ATOM 741 CG PRO R 603 62.618 86.979 78.022 1.00 1.00 C

ATOM 742 C PRO R 603 65.744 86.086 79.776 1.00 1.00 C

ATOM 743 O PRO R 603 66.427 87.033 79.364 1.00 1.00 O

ATOM 744 N CYS R 604 66.300 84.984 80.300 1.00 1.00 N

ATOM 745 CA CYS R 604 67.757 84.745 80.245 1.00 1.00 C

ATOM 746 CB CYS R 604 68.096 83.457 81.012 1.00 1.00 C

ATOM 747 SG CYS R 604 67.663 83.625 82.778 1.00 1.00 S

ATOM 748 C CYS R 604 68.262 84.695 78.762 1.00 1.00 C

ATOM 749 O CYS R 604 67.699 83.915 77.983 1.00 1.00 O

ATOM 750 N PRO R 605 69.267 85.496 78.304 1.00 1.00 N

ATOM 751 CA PRO R 605 69.779 85.394 76.906 1.00 1.00 C

ATOM 752 CD PRO R 605 70.026 86.454 79.129 1.00 1.00 C

ATOM 753 CB PRO R 605 70.773 86.573 76.830 1.00 1.00 C

ATOM 754 CG PRO R 605 70.531 87.431 78.076 1.00 1.00 C

ATOM 755 C PRO R 605 70.418 84.029 76.474 1.00 1.00 C

ATOM 756 O PRO R 605 70.508 83.742 75.279 1.00 1.00 O

ATOM 757 N ILE R 606 70.865 83.217 77.448 1.00 1.00 N

ATOM 758 CA ILE R 606 71.432 81.863 77.216 1.00 1.00 C

ATOM 759 CB ILE R 606 72.812 81.772 77.968 1.00 1.00 C

ATOM 760 CG2 ILE R 606 73.312 80.320 78.177 1.00 1.00 C

ATOM 761 CG1 ILE R 606 73.884 82.601 77.190 1.00 1.00 C

ATOM 762 CD1 ILE R 606 75.219 82.859 77.903 1.00 1.00 C

ATOM 763 C ILE R 606 70.337 80.784 77.534 1.00 1.00 C

ATOM 764 O ILE R 606 69.567 80.888 78.494 1.00 1.00 O

ATOM 765 N ASN R 607 70.298 79.770 76.653 1.00 1.00 N

ATOM 766 CA ASN R 607 69.139 78.857 76.467 1.00 1.00 C

ATOM 767 CB ASN R 607 69.455 77.984 75.212 1.00 1.00 C

ATOM 768 CG ASN R 607 68.861 78.500 73.897 1.00 1.00 C

ATOM 769 OD1 ASN R 607 67.668 78.758 73.775 1.00 1.00 O

ATOM 770 ND2 ASN R 607 69.622 78.593 72.835 1.00 1.00 N

ATOM 771 C ASN R 607 68.730 77.978 77.679 1.00 1.00 C

ATOM 772 O ASN R 607 69.500 77.084 78.103 1.00 1.00 O

ATOM 773 OXT ASN R 607 67.595 78.159 78.173 1.00 1.00 O

ATOM 774 N SER A 12 45.384 121.529 110.719 1.00 1.00 N

ATOM 775 CA SER A 12 46.517 120.739 111.279 1.00 1.00 C

ATOM 776 C SER A 12 46.171 120.036 112.635 1.00 1.00 C

ATOM 777 O SER A 12 45.943 118.826 112.606 1.00 1.00 O

ATOM 778 CB SER A 12 47.783 121.624 111.277 1.00 1.00 C

ATOM 779 OG SER A 12 47.614 122.803 112.070 1.00 1.00 O

ATOM 780 N ASP A 13 46.029 120.744 113.782 1.00 1.00 N

ATOM 781 CA ASP A 13 45.454 120.151 115.038 1.00 1.00 C

ATOM 782 C ASP A 13 43.977 119.645 114.856 1.00 1.00 C

ATOM 783 O ASP A 13 43.716 118.439 114.919 1.00 1.00 O

ATOM 784 CB ASP A 13 45.641 121.164 116.202 1.00 1.00 C

ATOM 785 CG ASP A 13 45.254 120.608 117.577 1.00 1.00 C

ATOM 786 OD1 ASP A 13 44.045 120.572 117.898 1.00 1.00 O

ATOM 787 OD2 ASP A 13 46.155 120.180 118.327 1.00 1.00 O

ATOM 788 N LEU A 14 43.045 120.554 114.506 1.00 1.00 N

ATOM 789 CA LEU A 14 41.675 120.174 114.055 1.00 1.00 C

ATOM 790 C LEU A 14 41.588 119.310 112.741 1.00 1.00 C

ATOM 791 O LEU A 14 40.634 118.547 112.587 1.00 1.00 O

ATOM 792 CB LEU A 14 40.773 121.434 113.937 1.00 1.00 C

ATOM 793 CG LEU A 14 40.617 122.418 115.131 1.00 1.00 C

ATOM 794 CD1 LEU A 14 39.439 123.373 114.854 1.00 1.00 C

ATOM 795 CD2 LEU A 14 40.409 121.735 116.494 1.00 1.00 C

ATOM 796 N GLY A 15 42.586 119.391 111.836 1.00 1.00 N

ATOM 797 CA GLY A 15 42.762 118.396 110.735 1.00 1.00 C

ATOM 798 C GLY A 15 43.078 116.937 111.150 1.00 1.00 C

ATOM 799 O GLY A 15 42.400 116.021 110.685 1.00 1.00 O

ATOM 800 N LYS A 16 44.057 116.721 112.052 1.00 1.00 N

ATOM 801 CA LYS A 16 44.245 115.417 112.758 1.00 1.00 C

ATOM 802 C LYS A 16 42.978 114.876 113.506 1.00 1.00 C

ATOM 803 O LYS A 16 42.586 113.733 113.261 1.00 1.00 O

ATOM 804 CB LYS A 16 45.490 115.539 113.683 1.00 1.00 C

ATOM 805 CG LYS A 16 45.962 114.185 114.266 1.00 1.00 C

ATOM 806 CD LYS A 16 47.195 114.304 115.180 1.00 1.00 C

ATOM 807 CE LYS A 16 47.619 112.921 115.709 1.00 1.00 C

ATOM 808 NZ LYS A 16 48.846 113.053 116.540 1.00 1.00 N

ATOM 809 N LYS A 17 42.315 115.699 114.344 1.00 1.00 N

ATOM 810 CA LYS A 17 40.974 115.364 114.917 1.00 1.00 C

ATOM 811 C LYS A 17 39.856 115.014 113.873 1.00 1.00 C

ATOM 812 O LYS A 17 39.093 114.074 114.097 1.00 1.00 O

ATOM 813 CB LYS A 17 40.502 116.520 115.843 1.00 1.00 C

ATOM 814 CG LYS A 17 41.374 116.780 117.092 1.00 1.00 C

ATOM 815 CD LYS A 17 40.833 117.944 117.953 1.00 1.00 C

ATOM 816 CE LYS A 17 41.720 118.317 119.157 1.00 1.00 C

ATOM 817 NZ LYS A 17 41.674 117.251 120.198 1.00 1.00 N

ATOM 818 N LEU A 18 39.768 115.735 112.738 1.00 1.00 N

ATOM 819 CA LEU A 18 38.854 115.387 111.614 1.00 1.00 C

ATOM 820 C LEU A 18 39.162 114.042 110.886 1.00 1.00 C

ATOM 821 O LEU A 18 38.243 113.255 110.650 1.00 1.00 O

ATOM 822 CB LEU A 18 38.828 116.609 110.659 1.00 1.00 C

ATOM 823 CG LEU A 18 37.800 116.536 109.507 1.00 1.00 C

ATOM 824 CD1 LEU A 18 36.352 116.586 110.014 1.00 1.00 C

ATOM 825 CD2 LEU A 18 38.058 117.671 108.515 1.00 1.00 C

ATOM 826 N LEU A 19 40.435 113.763 110.567 1.00 1.00 N

ATOM 827 CA LEU A 19 40.908 112.402 110.181 1.00 1.00 C

ATOM 828 C LEU A 19 40.540 111.257 111.185 1.00 1.00 C

ATOM 829 O LEU A 19 39.980 110.241 110.771 1.00 1.00 O

ATOM 830 CB LEU A 19 42.444 112.503 109.947 1.00 1.00 C

ATOM 831 CG LEU A 19 42.887 113.322 108.709 1.00 1.00 C

ATOM 832 CD1 LEU A 19 44.342 113.797 108.834 1.00 1.00 C

ATOM 833 CD2 LEU A 19 42.686 112.502 107.431 1.00 1.00 C

ATOM 834 N GLU A 20 40.803 111.441 112.491 1.00 1.00 N

ATOM 835 CA GLU A 20 40.304 110.549 113.581 1.00 1.00 C

ATOM 836 C GLU A 20 38.741 110.377 113.642 1.00 1.00 C

ATOM 837 O GLU A 20 38.264 109.238 113.615 1.00 1.00 O

ATOM 838 CB GLU A 20 40.935 111.055 114.913 1.00 1.00 C

ATOM 839 CG GLU A 20 42.482 110.895 115.032 1.00 1.00 C

ATOM 840 CD GLU A 20 43.219 111.700 116.100 1.00 1.00 C

ATOM 841 OE1 GLU A 20 42.599 112.438 116.896 1.00 1.00 O

ATOM 842 OE2 GLU A 20 44.466 111.605 116.116 1.00 1.00 O

ATOM 843 N ALA A 21 37.952 111.471 113.645 1.00 1.00 N

ATOM 844 CA ALA A 21 36.467 111.418 113.522 1.00 1.00 C

ATOM 845 C ALA A 21 35.877 110.769 112.217 1.00 1.00 C

ATOM 846 O ALA A 21 34.936 109.976 112.311 1.00 1.00 O

ATOM 847 CB ALA A 21 35.965 112.858 113.749 1.00 1.00 C

ATOM 848 N ALA A 22 36.440 111.052 111.022 1.00 1.00 N

ATOM 849 CA ALA A 22 36.137 110.301 109.771 1.00 1.00 C

ATOM 850 C ALA A 22 36.512 108.779 109.749 1.00 1.00 C

ATOM 851 O ALA A 22 35.708 107.973 109.273 1.00 1.00 O

ATOM 852 CB ALA A 22 36.795 111.076 108.615 1.00 1.00 C

ATOM 853 N ARG A 23 37.682 108.369 110.288 1.00 1.00 N

ATOM 854 CA ARG A 23 37.987 106.932 110.568 1.00 1.00 C

ATOM 855 C ARG A 23 37.010 106.231 111.572 1.00 1.00 C

ATOM 856 O ARG A 23 36.470 105.170 111.253 1.00 1.00 O

ATOM 857 CB ARG A 23 39.450 106.758 111.067 1.00 1.00 C

ATOM 858 CG ARG A 23 40.564 107.045 110.040 1.00 1.00 C

ATOM 859 CD ARG A 23 41.961 106.618 110.537 1.00 1.00 C

ATOM 860 NE ARG A 23 43.030 107.447 109.908 1.00 1.00 N

ATOM 861 CZ ARG A 23 43.609 108.509 110.467 1.00 1.00 C

ATOM 862 NH1 ARG A 23 43.297 108.976 111.651 1.00 1.00 N

ATOM 863 NH2 ARG A 23 44.533 109.126 109.805 1.00 1.00 N

ATOM 864 N ALA A 24 36.795 106.800 112.773 1.00 1.00 N

ATOM 865 CA ALA A 24 35.906 106.203 113.805 1.00 1.00 C

ATOM 866 C ALA A 24 34.352 106.335 113.630 1.00 1.00 C

ATOM 867 O ALA A 24 33.616 105.907 114.525 1.00 1.00 O

ATOM 868 CB ALA A 24 36.387 106.845 115.123 1.00 1.00 C

ATOM 869 N GLY A 25 33.844 106.916 112.527 1.00 1.00 N

ATOM 870 CA GLY A 25 32.389 107.189 112.348 1.00 1.00 C

ATOM 871 C GLY A 25 31.713 108.157 113.337 1.00 1.00 C

ATOM 872 O GLY A 25 30.601 107.886 113.793 1.00 1.00 O

ATOM 873 N GLN A 26 32.373 109.278 113.658 1.00 1.00 N

ATOM 874 CA GLN A 26 31.865 110.241 114.667 1.00 1.00 C

ATOM 875 C GLN A 26 31.205 111.451 113.941 1.00 1.00 C

ATOM 876 O GLN A 26 31.803 112.523 113.794 1.00 1.00 O

ATOM 877 CB GLN A 26 33.011 110.625 115.644 1.00 1.00 C

ATOM 878 CG GLN A 26 33.710 109.486 116.434 1.00 1.00 C

ATOM 879 CD GLN A 26 32.811 108.652 117.356 1.00 1.00 C

ATOM 880 OE1 GLN A 26 32.076 109.158 118.197 1.00 1.00 O

ATOM 881 NE2 GLN A 26 32.831 107.345 117.242 1.00 1.00 N

ATOM 882 N ASP A 27 29.946 111.257 113.502 1.00 1.00 N

ATOM 883 CA ASP A 27 29.169 112.262 112.716 1.00 1.00 C

ATOM 884 C ASP A 27 29.081 113.697 113.337 1.00 1.00 C

ATOM 885 O ASP A 27 29.373 114.695 112.670 1.00 1.00 O

ATOM 886 CB ASP A 27 27.749 111.683 112.444 1.00 1.00 C

ATOM 887 CG ASP A 27 27.594 110.417 111.594 1.00 1.00 C

ATOM 888 OD1 ASP A 27 28.594 109.756 111.244 1.00 1.00 O

ATOM 889 OD2 ASP A 27 26.435 110.078 111.282 1.00 1.00 O

ATOM 890 N ASP A 28 28.728 113.779 114.628 1.00 1.00 N

ATOM 891 CA ASP A 28 28.650 115.059 115.378 1.00 1.00 C

ATOM 892 C ASP A 28 30.029 115.693 115.778 1.00 1.00 C

ATOM 893 O ASP A 28 30.146 116.919 115.715 1.00 1.00 O

ATOM 894 CB ASP A 28 27.656 114.859 116.550 1.00 1.00 C

ATOM 895 CG ASP A 28 26.190 114.609 116.139 1.00 1.00 C

ATOM 896 OD1 ASP A 28 25.761 115.036 115.036 1.00 1.00 O

ATOM 897 OD2 ASP A 28 25.468 113.966 116.928 1.00 1.00 O

ATOM 898 N GLU A 29 31.093 114.911 116.080 1.00 1.00 N

ATOM 899 CA GLU A 29 32.504 115.420 116.064 1.00 1.00 C

ATOM 900 C GLU A 29 32.954 116.058 114.703 1.00 1.00 C

ATOM 901 O GLU A 29 33.461 117.183 114.707 1.00 1.00 O

ATOM 902 CB GLU A 29 33.512 114.319 116.489 1.00 1.00 C

ATOM 903 CG GLU A 29 33.445 113.828 117.958 1.00 1.00 C

ATOM 904 CD GLU A 29 34.473 112.773 118.366 1.00 1.00 C

ATOM 905 OE1 GLU A 29 35.409 112.461 117.596 1.00 1.00 O

ATOM 906 OE2 GLU A 29 34.338 112.253 119.491 1.00 1.00 O

ATOM 907 N VAL A 30 32.716 115.394 113.547 1.00 1.00 N

ATOM 908 CA VAL A 30 32.850 116.019 112.189 1.00 1.00 C

ATOM 909 C VAL A 30 32.053 117.368 112.041 1.00 1.00 C

ATOM 910 O VAL A 30 32.662 118.380 111.686 1.00 1.00 O

ATOM 911 CB VAL A 30 32.537 114.967 111.061 1.00 1.00 C

ATOM 912 CG1 VAL A 30 32.523 115.552 109.627 1.00 1.00 C

ATOM 913 CG2 VAL A 30 33.514 113.765 111.048 1.00 1.00 C

ATOM 914 N ARG A 31 30.739 117.406 112.343 1.00 1.00 N

ATOM 915 CA ARG A 31 29.943 118.674 112.391 1.00 1.00 C

ATOM 916 C ARG A 31 30.496 119.809 113.328 1.00 1.00 C

ATOM 917 O ARG A 31 30.590 120.958 112.886 1.00 1.00 O

ATOM 918 CB ARG A 31 28.463 118.346 112.731 1.00 1.00 C

ATOM 919 CG ARG A 31 27.695 117.518 111.673 1.00 1.00 C

ATOM 920 CD ARG A 31 26.248 117.213 112.100 1.00 1.00 C

ATOM 921 NE ARG A 31 25.586 116.441 111.016 1.00 1.00 N

ATOM 922 CZ ARG A 31 25.166 115.178 111.085 1.00 1.00 C

ATOM 923 NH1 ARG A 31 25.183 114.432 112.167 1.00 1.00 N

ATOM 924 NH2 ARG A 31 24.694 114.662 109.987 1.00 1.00 N

ATOM 925 N ILE A 32 30.902 119.498 114.578 1.00 1.00 N

ATOM 926 CA ILE A 32 31.656 120.441 115.473 1.00 1.00 C

ATOM 927 C ILE A 32 32.997 120.964 114.837 1.00 1.00 C

ATOM 928 O ILE A 32 33.233 122.175 114.819 1.00 1.00 O

ATOM 929 CB ILE A 32 31.842 119.799 116.903 1.00 1.00 C

ATOM 930 CG1 ILE A 32 30.493 119.531 117.643 1.00 1.00 C

ATOM 931 CG2 ILE A 32 32.755 120.634 117.847 1.00 1.00 C

ATOM 932 CD1 ILE A 32 30.571 118.497 118.783 1.00 1.00 C

ATOM 933 N LEU A 33 33.861 120.073 114.325 1.00 1.00 N

ATOM 934 CA LEU A 33 35.108 120.450 113.597 1.00 1.00 C

ATOM 935 C LEU A 33 34.901 121.297 112.293 1.00 1.00 C

ATOM 936 O LEU A 33 35.613 122.287 112.103 1.00 1.00 O

ATOM 937 CB LEU A 33 35.912 119.140 113.360 1.00 1.00 C

ATOM 938 CG LEU A 33 36.466 118.441 114.636 1.00 1.00 C

ATOM 939 CD1 LEU A 33 36.822 116.986 114.323 1.00 1.00 C

ATOM 940 CD2 LEU A 33 37.697 119.161 115.212 1.00 1.00 C

ATOM 941 N MET A 34 33.904 120.979 111.444 1.00 1.00 N

ATOM 942 CA MET A 34 33.414 121.888 110.363 1.00 1.00 C

ATOM 943 C MET A 34 32.931 123.300 110.839 1.00 1.00 C

ATOM 944 O MET A 34 33.428 124.309 110.331 1.00 1.00 O

ATOM 945 CB MET A 34 32.309 121.160 109.551 1.00 1.00 C

ATOM 946 CG MET A 34 32.753 119.941 108.717 1.00 1.00 C

ATOM 947 SD MET A 34 33.965 120.415 107.468 1.00 1.00 S

ATOM 948 CE MET A 34 35.479 119.847 108.240 1.00 1.00 C

ATOM 949 N ALA A 35 32.033 123.384 111.843 1.00 1.00 N

ATOM 950 CA ALA A 35 31.697 124.664 112.532 1.00 1.00 C

ATOM 951 C ALA A 35 32.875 125.499 113.151 1.00 1.00 C

ATOM 952 O ALA A 35 32.808 126.730 113.159 1.00 1.00 O

ATOM 953 CB ALA A 35 30.641 124.306 113.595 1.00 1.00 C

ATOM 954 N ASN A 36 33.948 124.847 113.633 1.00 1.00 N

ATOM 955 CA ASN A 36 35.195 125.530 114.099 1.00 1.00 C

ATOM 956 C ASN A 36 36.329 125.721 113.024 1.00 1.00 C

ATOM 957 O ASN A 36 37.463 126.041 113.397 1.00 1.00 O

ATOM 958 CB ASN A 36 35.694 124.734 115.343 1.00 1.00 C

ATOM 959 CG ASN A 36 34.877 124.947 116.623 1.00 1.00 C

ATOM 960 OD1 ASN A 36 35.129 125.854 117.407 1.00 1.00 O

ATOM 961 ND2 ASN A 36 33.872 124.144 116.873 1.00 1.00 N

ATOM 962 N GLY A 37 36.058 125.574 111.711 1.00 1.00 N

ATOM 963 CA GLY A 37 37.086 125.734 110.645 1.00 1.00 C

ATOM 964 C GLY A 37 38.267 124.738 110.598 1.00 1.00 C

ATOM 965 O GLY A 37 39.396 125.149 110.320 1.00 1.00 O

ATOM 966 N ALA A 38 38.010 123.434 110.791 1.00 1.00 N

ATOM 967 CA ALA A 38 39.009 122.373 110.521 1.00 1.00 C

ATOM 968 C ALA A 38 39.330 122.212 109.006 1.00 1.00 C

ATOM 969 O ALA A 38 38.456 122.388 108.148 1.00 1.00 O

ATOM 970 CB ALA A 38 38.442 121.064 111.099 1.00 1.00 C

ATOM 971 N ASP A 39 40.584 121.846 108.682 1.00 1.00 N

ATOM 972 CA ASP A 39 40.995 121.578 107.279 1.00 1.00 C

ATOM 973 C ASP A 39 40.265 120.319 106.702 1.00 1.00 C

ATOM 974 O ASP A 39 40.648 119.170 106.947 1.00 1.00 O

ATOM 975 CB ASP A 39 42.544 121.503 107.244 1.00 1.00 C

ATOM 976 CG ASP A 39 43.200 121.269 105.879 1.00 1.00 C

ATOM 977 OD1 ASP A 39 42.516 121.303 104.830 1.00 1.00 O

ATOM 978 OD2 ASP A 39 44.426 121.031 105.871 1.00 1.00 O

ATOM 979 N VAL A 40 39.211 120.577 105.909 1.00 1.00 N

ATOM 980 CA VAL A 40 38.442 119.524 105.179 1.00 1.00 C

ATOM 981 C VAL A 40 39.274 118.558 104.256 1.00 1.00 C

ATOM 982 O VAL A 40 38.897 117.395 104.090 1.00 1.00 O

ATOM 983 CB VAL A 40 37.218 120.213 104.479 1.00 1.00 C

ATOM 984 CG1 VAL A 40 37.561 120.938 103.157 1.00 1.00 C

ATOM 985 CG2 VAL A 40 36.055 119.231 104.242 1.00 1.00 C

ATOM 986 N ASN A 41 40.398 119.042 103.695 1.00 1.00 N

ATOM 987 CA ASN A 41 41.361 118.208 102.923 1.00 1.00 C

ATOM 988 C ASN A 41 42.708 117.922 103.675 1.00 1.00 C

ATOM 989 O ASN A 41 43.747 117.734 103.031 1.00 1.00 O

ATOM 990 CB ASN A 41 41.563 118.914 101.550 1.00 1.00 C

ATOM 991 CG ASN A 41 40.295 118.995 100.692 1.00 1.00 C

ATOM 992 OD1 ASN A 41 39.570 118.018 100.550 1.00 1.00 O

ATOM 993 ND2 ASN A 41 39.961 120.146 100.155 1.00 1.00 N

ATOM 994 N ALA A 42 42.688 117.781 105.019 1.00 1.00 N

ATOM 995 CA ALA A 42 43.883 117.427 105.825 1.00 1.00 C

ATOM 996 C ALA A 42 44.448 116.022 105.478 1.00 1.00 C

ATOM 997 O ALA A 42 43.687 115.056 105.435 1.00 1.00 O

ATOM 998 CB ALA A 42 43.444 117.471 107.301 1.00 1.00 C

ATOM 999 N LYS A 43 45.758 115.901 105.215 1.00 1.00 N

ATOM 1000 CA LYS A 43 46.364 114.593 104.840 1.00 1.00 C

ATOM 1001 C LYS A 43 47.045 113.900 106.053 1.00 1.00 C

ATOM 1002 O LYS A 43 47.896 114.491 106.727 1.00 1.00 O

ATOM 1003 CB LYS A 43 47.318 114.729 103.629 1.00 1.00 C

ATOM 1004 CG LYS A 43 46.588 114.992 102.289 1.00 1.00 C

ATOM 1005 CD LYS A 43 47.538 114.879 101.083 1.00 1.00 C

ATOM 1006 CE LYS A 43 46.849 115.274 99.767 1.00 1.00 C

ATOM 1007 NZ LYS A 43 47.795 115.064 98.639 1.00 1.00 N

ATOM 1008 N ASP A 44 46.702 112.623 106.289 1.00 1.00 N

ATOM 1009 CA ASP A 44 47.455 111.755 107.232 1.00 1.00 C

ATOM 1010 C ASP A 44 48.842 111.262 106.683 1.00 1.00 C

ATOM 1011 O ASP A 44 49.215 111.498 105.528 1.00 1.00 O

ATOM 1012 CB ASP A 44 46.493 110.634 107.723 1.00 1.00 C

ATOM 1013 CG ASP A 44 46.047 109.507 106.789 1.00 1.00 C

ATOM 1014 OD1 ASP A 44 46.493 109.440 105.625 1.00 1.00 O

ATOM 1015 OD2 ASP A 44 45.243 108.665 107.256 1.00 1.00 O

ATOM 1016 N GLU A 45 49.605 110.538 107.521 1.00 1.00 N

ATOM 1017 CA GLU A 45 50.840 109.809 107.086 1.00 1.00 C

ATOM 1018 C GLU A 45 50.712 108.790 105.890 1.00 1.00 C

ATOM 1019 O GLU A 45 51.655 108.602 105.118 1.00 1.00 O

ATOM 1020 CB GLU A 45 51.473 109.177 108.358 1.00 1.00 C

ATOM 1021 CG GLU A 45 50.772 107.910 108.928 1.00 1.00 C

ATOM 1022 CD GLU A 45 51.188 107.469 110.322 1.00 1.00 C

ATOM 1023 OE1 GLU A 45 52.394 107.483 110.645 1.00 1.00 O

ATOM 1024 OE2 GLU A 45 50.289 107.080 111.098 1.00 1.00 O

ATOM 1025 N TYR A 46 49.526 108.183 105.719 1.00 1.00 N

ATOM 1026 CA TYR A 46 49.138 107.407 104.506 1.00 1.00 C

ATOM 1027 C TYR A 46 48.803 108.267 103.220 1.00 1.00 C

ATOM 1028 O TYR A 46 48.509 107.693 102.168 1.00 1.00 O

ATOM 1029 CB TYR A 46 47.919 106.502 104.897 1.00 1.00 C

ATOM 1030 CG TYR A 46 48.038 105.562 106.124 1.00 1.00 C

ATOM 1031 CD1 TYR A 46 47.848 106.069 107.415 1.00 1.00 C

ATOM 1032 CD2 TYR A 46 48.380 104.217 105.970 1.00 1.00 C

ATOM 1033 CE1 TYR A 46 48.086 105.269 108.530 1.00 1.00 C

ATOM 1034 CE2 TYR A 46 48.607 103.411 107.083 1.00 1.00 C

ATOM 1035 CZ TYR A 46 48.475 103.943 108.361 1.00 1.00 C

ATOM 1036 OH TYR A 46 48.753 103.171 109.455 1.00 1.00 O

ATOM 1037 N GLY A 47 48.844 109.616 103.289 1.00 1.00 N

ATOM 1038 CA GLY A 47 48.470 110.528 102.179 1.00 1.00 C

ATOM 1039 C GLY A 47 46.999 110.990 102.057 1.00 1.00 C

ATOM 1040 O GLY A 47 46.655 111.504 100.989 1.00 1.00 O

ATOM 1041 N LEU A 48 46.126 110.827 103.072 1.00 1.00 N

ATOM 1042 CA LEU A 48 44.658 110.765 102.827 1.00 1.00 C

ATOM 1043 C LEU A 48 43.805 111.877 103.452 1.00 1.00 C

ATOM 1044 O LEU A 48 43.997 112.273 104.598 1.00 1.00 O

ATOM 1045 CB LEU A 48 44.066 109.431 103.341 1.00 1.00 C

ATOM 1046 CG LEU A 48 44.587 108.124 102.724 1.00 1.00 C

ATOM 1047 CD1 LEU A 48 43.666 106.994 103.237 1.00 1.00 C

ATOM 1048 CD2 LEU A 48 44.707 108.165 101.182 1.00 1.00 C

ATOM 1049 N THR A 49 42.764 112.253 102.699 1.00 1.00 N

ATOM 1050 CA THR A 49 41.722 113.198 103.152 1.00 1.00 C

ATOM 1051 C THR A 49 40.588 112.491 103.987 1.00 1.00 C

ATOM 1052 O THR A 49 40.278 111.318 103.728 1.00 1.00 O

ATOM 1053 CB THR A 49 41.135 113.957 101.918 1.00 1.00 C

ATOM 1054 OG1 THR A 49 40.445 113.074 101.035 1.00 1.00 O

ATOM 1055 CG2 THR A 49 42.173 114.729 101.080 1.00 1.00 C

ATOM 1056 N PRO A 50 39.879 113.181 104.932 1.00 1.00 N

ATOM 1057 CA PRO A 50 38.598 112.685 105.521 1.00 1.00 C

ATOM 1058 C PRO A 50 37.502 112.097 104.569 1.00 1.00 C

ATOM 1059 O PRO A 50 36.839 111.125 104.934 1.00 1.00 O

ATOM 1060 CB PRO A 50 38.100 113.927 106.289 1.00 1.00 C

ATOM 1061 CG PRO A 50 39.354 114.730 106.621 1.00 1.00 C

ATOM 1062 CD PRO A 50 40.259 114.520 105.419 1.00 1.00 C

ATOM 1063 N LEU A 51 37.329 112.669 103.360 1.00 1.00 N

ATOM 1064 CA LEU A 51 36.386 112.151 102.326 1.00 1.00 C

ATOM 1065 C LEU A 51 36.693 110.717 101.791 1.00 1.00 C

ATOM 1066 O LEU A 51 35.765 109.911 101.681 1.00 1.00 O

ATOM 1067 CB LEU A 51 36.313 113.208 101.188 1.00 1.00 C

ATOM 1068 CG LEU A 51 35.291 112.940 100.052 1.00 1.00 C

ATOM 1069 CD1 LEU A 51 33.840 113.018 100.548 1.00 1.00 C

ATOM 1070 CD2 LEU A 51 35.504 113.917 98.886 1.00 1.00 C

ATOM 1071 N TYR A 52 37.964 110.399 101.482 1.00 1.00 N

ATOM 1072 CA TYR A 52 38.410 109.002 101.224 1.00 1.00 C

ATOM 1073 C TYR A 52 38.169 108.013 102.423 1.00 1.00 C

ATOM 1074 O TYR A 52 37.584 106.946 102.225 1.00 1.00 O

ATOM 1075 CB TYR A 52 39.894 109.092 100.759 1.00 1.00 C

ATOM 1076 CG TYR A 52 40.427 107.800 100.123 1.00 1.00 C

ATOM 1077 CD1 TYR A 52 40.908 106.763 100.929 1.00 1.00 C

ATOM 1078 CD2 TYR A 52 40.385 107.628 98.735 1.00 1.00 C

ATOM 1079 CE1 TYR A 52 41.318 105.563 100.354 1.00 1.00 C

ATOM 1080 CE2 TYR A 52 40.801 106.427 98.166 1.00 1.00 C

ATOM 1081 CZ TYR A 52 41.260 105.395 98.974 1.00 1.00 C

ATOM 1082 OH TYR A 52 41.658 104.225 98.389 1.00 1.00 O

ATOM 1083 N LEU A 53 38.587 108.375 103.650 1.00 1.00 N

ATOM 1084 CA LEU A 53 38.371 107.549 104.877 1.00 1.00 C

ATOM 1085 C LEU A 53 36.876 107.330 105.299 1.00 1.00 C

ATOM 1086 O LEU A 53 36.488 106.193 105.581 1.00 1.00 O

ATOM 1087 CB LEU A 53 39.237 108.158 106.018 1.00 1.00 C

ATOM 1088 CG LEU A 53 40.779 108.069 105.826 1.00 1.00 C

ATOM 1089 CD1 LEU A 53 41.512 109.043 106.758 1.00 1.00 C

ATOM 1090 CD2 LEU A 53 41.313 106.637 106.021 1.00 1.00 C

ATOM 1091 N ALA A 54 36.026 108.375 105.291 1.00 1.00 N

ATOM 1092 CA ALA A 54 34.548 108.220 105.385 1.00 1.00 C

ATOM 1093 C ALA A 54 33.848 107.423 104.234 1.00 1.00 C

ATOM 1094 O ALA A 54 32.987 106.586 104.514 1.00 1.00 O

ATOM 1095 CB ALA A 54 33.984 109.642 105.501 1.00 1.00 C

ATOM 1096 N THR A 55 34.242 107.647 102.963 1.00 1.00 N

ATOM 1097 CA THR A 55 33.850 106.780 101.806 1.00 1.00 C

ATOM 1098 C THR A 55 34.180 105.264 101.996 1.00 1.00 C

ATOM 1099 O THR A 55 33.283 104.431 101.855 1.00 1.00 O

ATOM 1100 CB THR A 55 34.473 107.364 100.501 1.00 1.00 C

ATOM 1101 OG1 THR A 55 34.027 108.700 100.281 1.00 1.00 O

ATOM 1102 CG2 THR A 55 34.164 106.587 99.218 1.00 1.00 C

ATOM 1103 N ALA A 56 35.437 104.927 102.342 1.00 1.00 N

ATOM 1104 CA ALA A 56 35.871 103.547 102.681 1.00 1.00 C

ATOM 1105 C ALA A 56 34.989 102.731 103.669 1.00 1.00 C

ATOM 1106 O ALA A 56 34.602 101.603 103.364 1.00 1.00 O

ATOM 1107 CB ALA A 56 37.287 103.698 103.264 1.00 1.00 C

ATOM 1108 N HIS A 57 34.666 103.340 104.820 1.00 1.00 N

ATOM 1109 CA HIS A 57 33.774 102.737 105.844 1.00 1.00 C

ATOM 1110 C HIS A 57 32.225 102.868 105.595 1.00 1.00 C

ATOM 1111 O HIS A 57 31.434 102.594 106.502 1.00 1.00 O

ATOM 1112 CB HIS A 57 34.166 103.412 107.186 1.00 1.00 C

ATOM 1113 CG HIS A 57 35.552 103.127 107.766 1.00 1.00 C

ATOM 1114 ND1 HIS A 57 35.968 101.870 108.177 1.00 1.00 N

ATOM 1115 CD2 HIS A 57 36.508 104.100 108.101 1.00 1.00 C

ATOM 1116 CE1 HIS A 57 37.174 102.207 108.726 1.00 1.00 C

ATOM 1117 NE2 HIS A 57 37.600 103.508 108.711 1.00 1.00 N

ATOM 1118 N GLY A 58 31.765 103.327 104.413 1.00 1.00 N

ATOM 1119 CA GLY A 58 30.328 103.615 104.169 1.00 1.00 C

ATOM 1120 C GLY A 58 29.623 104.711 105.010 1.00 1.00 C

ATOM 1121 O GLY A 58 28.406 104.629 105.196 1.00 1.00 O

ATOM 1122 N HIS A 59 30.342 105.752 105.470 1.00 1.00 N

ATOM 1123 CA HIS A 59 29.773 106.786 106.380 1.00 1.00 C

ATOM 1124 C HIS A 59 29.133 107.946 105.556 1.00 1.00 C

ATOM 1125 O HIS A 59 29.739 109.001 105.337 1.00 1.00 O

ATOM 1126 CB HIS A 59 30.855 107.273 107.389 1.00 1.00 C

ATOM 1127 CG HIS A 59 31.385 106.252 108.405 1.00 1.00 C

ATOM 1128 ND1 HIS A 59 30.608 105.284 109.031 1.00 1.00 N

ATOM 1129 CD2 HIS A 59 32.717 106.175 108.847 1.00 1.00 C

ATOM 1130 CE1 HIS A 59 31.578 104.662 109.771 1.00 1.00 C

ATOM 1131 NE2 HIS A 59 32.864 105.133 109.745 1.00 1.00 N

ATOM 1132 N LEU A 60 27.880 107.725 105.113 1.00 1.00 N

ATOM 1133 CA LEU A 60 27.120 108.686 104.260 1.00 1.00 C

ATOM 1134 C LEU A 60 26.953 110.120 104.855 1.00 1.00 C

ATOM 1135 O LEU A 60 27.388 111.079 104.217 1.00 1.00 O

ATOM 1136 CB LEU A 60 25.784 107.993 103.863 1.00 1.00 C

ATOM 1137 CG LEU A 60 24.783 108.814 103.003 1.00 1.00 C

ATOM 1138 CD1 LEU A 60 25.339 109.178 101.616 1.00 1.00 C

ATOM 1139 CD2 LEU A 60 23.463 108.042 102.854 1.00 1.00 C

ATOM 1140 N GLU A 61 26.391 110.253 106.071 1.00 1.00 N

ATOM 1141 CA GLU A 61 26.316 111.543 106.824 1.00 1.00 C

ATOM 1142 C GLU A 61 27.652 112.370 106.908 1.00 1.00 C

ATOM 1143 O GLU A 61 27.658 113.566 106.603 1.00 1.00 O

ATOM 1144 CB GLU A 61 25.769 111.207 108.242 1.00 1.00 C

ATOM 1145 CG GLU A 61 24.283 110.756 108.355 1.00 1.00 C

ATOM 1146 CD GLU A 61 23.286 111.841 108.729 1.00 1.00 C

ATOM 1147 OE1 GLU A 61 23.406 112.405 109.841 1.00 1.00 O

ATOM 1148 OE2 GLU A 61 22.374 112.134 107.931 1.00 1.00 O

ATOM 1149 N ILE A 62 28.789 111.724 107.245 1.00 1.00 N

ATOM 1150 CA ILE A 62 30.154 112.336 107.153 1.00 1.00 C

ATOM 1151 C ILE A 62 30.544 112.750 105.690 1.00 1.00 C

ATOM 1152 O ILE A 62 30.906 113.912 105.495 1.00 1.00 O

ATOM 1153 CB ILE A 62 31.216 111.435 107.885 1.00 1.00 C

ATOM 1154 CG1 ILE A 62 30.938 111.320 109.410 1.00 1.00 C

ATOM 1155 CG2 ILE A 62 32.674 111.919 107.683 1.00 1.00 C

ATOM 1156 CD1 ILE A 62 31.734 110.246 110.166 1.00 1.00 C

ATOM 1157 N VAL A 63 30.461 111.859 104.678 1.00 1.00 N

ATOM 1158 CA VAL A 63 30.615 112.233 103.229 1.00 1.00 C

ATOM 1159 C VAL A 63 29.758 113.482 102.794 1.00 1.00 C

ATOM 1160 O VAL A 63 30.333 114.458 102.308 1.00 1.00 O

ATOM 1161 CB VAL A 63 30.405 110.965 102.321 1.00 1.00 C

ATOM 1162 CG1 VAL A 63 30.340 111.256 100.803 1.00 1.00 C

ATOM 1163 CG2 VAL A 63 31.492 109.880 102.509 1.00 1.00 C

ATOM 1164 N GLU A 64 28.428 113.473 103.012 1.00 1.00 N

ATOM 1165 CA GLU A 64 27.543 114.659 102.805 1.00 1.00 C

ATOM 1166 C GLU A 64 28.000 115.984 103.517 1.00 1.00 C

ATOM 1167 O GLU A 64 28.114 117.024 102.861 1.00 1.00 O

ATOM 1168 CB GLU A 64 26.089 114.307 103.230 1.00 1.00 C

ATOM 1169 CG GLU A 64 25.370 113.148 102.492 1.00 1.00 C

ATOM 1170 CD GLU A 64 23.891 113.046 102.815 1.00 1.00 C

ATOM 1171 OE1 GLU A 64 23.543 112.850 103.996 1.00 1.00 O

ATOM 1172 OE2 GLU A 64 23.071 113.182 101.883 1.00 1.00 O

ATOM 1173 N VAL A 65 28.284 115.939 104.835 1.00 1.00 N

ATOM 1174 CA VAL A 65 28.876 117.079 105.609 1.00 1.00 C

ATOM 1175 C VAL A 65 30.258 117.578 105.052 1.00 1.00 C

ATOM 1176 O VAL A 65 30.410 118.778 104.805 1.00 1.00 O

ATOM 1177 CB VAL A 65 28.887 116.702 107.138 1.00 1.00 C

ATOM 1178 CG1 VAL A 65 29.668 117.679 108.046 1.00 1.00 C

ATOM 1179 CG2 VAL A 65 27.472 116.580 107.753 1.00 1.00 C

ATOM 1180 N LEU A 66 31.242 116.686 104.844 1.00 1.00 N

ATOM 1181 CA LEU A 66 32.539 117.023 104.188 1.00 1.00 C

ATOM 1182 C LEU A 66 32.420 117.653 102.759 1.00 1.00 C

ATOM 1183 O LEU A 66 32.995 118.717 102.520 1.00 1.00 O

ATOM 1184 CB LEU A 66 33.426 115.747 104.176 1.00 1.00 C

ATOM 1185 CG LEU A 66 33.892 115.174 105.540 1.00 1.00 C

ATOM 1186 CD1 LEU A 66 34.548 113.813 105.293 1.00 1.00 C

ATOM 1187 CD2 LEU A 66 34.882 116.092 106.275 1.00 1.00 C

ATOM 1188 N LEU A 67 31.654 117.041 101.836 1.00 1.00 N

ATOM 1189 CA LEU A 67 31.324 117.638 100.508 1.00 1.00 C

ATOM 1190 C LEU A 67 30.630 119.041 100.545 1.00 1.00 C

ATOM 1191 O LEU A 67 31.100 119.967 99.881 1.00 1.00 O

ATOM 1192 CB LEU A 67 30.473 116.614 99.709 1.00 1.00 C

ATOM 1193 CG LEU A 67 31.151 115.296 99.267 1.00 1.00 C

ATOM 1194 CD1 LEU A 67 30.102 114.339 98.680 1.00 1.00 C

ATOM 1195 CD2 LEU A 67 32.274 115.538 98.249 1.00 1.00 C

ATOM 1196 N LYS A 68 29.572 119.224 101.361 1.00 1.00 N

ATOM 1197 CA LYS A 68 29.016 120.574 101.694 1.00 1.00 C

ATOM 1198 C LYS A 68 30.025 121.645 102.238 1.00 1.00 C

ATOM 1199 O LYS A 68 29.883 122.826 101.915 1.00 1.00 O

ATOM 1200 CB LYS A 68 27.835 120.374 102.686 1.00 1.00 C

ATOM 1201 CG LYS A 68 26.552 119.777 102.059 1.00 1.00 C

ATOM 1202 CD LYS A 68 25.515 119.363 103.123 1.00 1.00 C

ATOM 1203 CE LYS A 68 24.235 118.793 102.485 1.00 1.00 C

ATOM 1204 NZ LYS A 68 23.342 118.235 103.537 1.00 1.00 N

ATOM 1205 N ASN A 69 31.037 121.243 103.024 1.00 1.00 N

ATOM 1206 CA ASN A 69 32.149 122.143 103.457 1.00 1.00 C

ATOM 1207 C ASN A 69 33.409 122.194 102.511 1.00 1.00 C

ATOM 1208 O ASN A 69 34.470 122.654 102.940 1.00 1.00 O

ATOM 1209 CB ASN A 69 32.503 121.719 104.911 1.00 1.00 C

ATOM 1210 CG ASN A 69 31.463 122.100 105.974 1.00 1.00 C

ATOM 1211 OD1 ASN A 69 31.490 123.183 106.547 1.00 1.00 O

ATOM 1212 ND2 ASN A 69 30.522 121.240 106.280 1.00 1.00 N

ATOM 1213 N GLY A 70 33.303 121.796 101.227 1.00 1.00 N

ATOM 1214 CA GLY A 70 34.421 121.885 100.245 1.00 1.00 C

ATOM 1215 C GLY A 70 35.488 120.767 100.216 1.00 1.00 C

ATOM 1216 O GLY A 70 36.645 121.059 99.902 1.00 1.00 O

ATOM 1217 N ALA A 71 35.121 119.499 100.470 1.00 1.00 N

ATOM 1218 CA ALA A 71 36.060 118.359 100.332 1.00 1.00 C

ATOM 1219 C ALA A 71 36.385 117.998 98.852 1.00 1.00 C

ATOM 1220 O ALA A 71 35.503 117.954 97.986 1.00 1.00 O

ATOM 1221 CB ALA A 71 35.465 117.148 101.061 1.00 1.00 C

ATOM 1222 N ASP A 72 37.668 117.718 98.584 1.00 1.00 N

ATOM 1223 CA ASP A 72 38.174 117.440 97.224 1.00 1.00 C

ATOM 1224 C ASP A 72 37.757 116.016 96.735 1.00 1.00 C

ATOM 1225 O ASP A 72 38.381 114.994 97.039 1.00 1.00 O

ATOM 1226 CB ASP A 72 39.705 117.694 97.234 1.00 1.00 C

ATOM 1227 CG ASP A 72 40.423 117.589 95.887 1.00 1.00 C

ATOM 1228 OD1 ASP A 72 39.761 117.615 94.823 1.00 1.00 O

ATOM 1229 OD2 ASP A 72 41.666 117.471 95.895 1.00 1.00 O

ATOM 1230 N VAL A 73 36.713 115.996 95.900 1.00 1.00 N

ATOM 1231 CA VAL A 73 36.355 114.826 95.037 1.00 1.00 C

ATOM 1232 C VAL A 73 37.486 114.272 94.099 1.00 1.00 C

ATOM 1233 O VAL A 73 37.478 113.079 93.787 1.00 1.00 O

ATOM 1234 CB VAL A 73 35.051 115.130 94.220 1.00 1.00 C

ATOM 1235 CG1 VAL A 73 33.798 115.362 95.090 1.00 1.00 C

ATOM 1236 CG2 VAL A 73 35.178 116.295 93.208 1.00 1.00 C

ATOM 1237 N ASN A 74 38.443 115.114 93.667 1.00 1.00 N

ATOM 1238 CA ASN A 74 39.655 114.673 92.920 1.00 1.00 C

ATOM 1239 C ASN A 74 40.854 114.171 93.805 1.00 1.00 C

ATOM 1240 O ASN A 74 41.878 113.784 93.234 1.00 1.00 O

ATOM 1241 CB ASN A 74 40.124 115.842 92.006 1.00 1.00 C

ATOM 1242 CG ASN A 74 39.087 116.437 91.040 1.00 1.00 C

ATOM 1243 OD1 ASN A 74 38.439 115.746 90.261 1.00 1.00 O

ATOM 1244 ND2 ASN A 74 38.882 117.732 91.082 1.00 1.00 N

ATOM 1245 N ALA A 75 40.758 114.137 95.156 1.00 1.00 N

ATOM 1246 CA ALA A 75 41.838 113.653 96.047 1.00 1.00 C

ATOM 1247 C ALA A 75 42.130 112.138 95.858 1.00 1.00 C

ATOM 1248 O ALA A 75 41.337 111.273 96.245 1.00 1.00 O

ATOM 1249 CB ALA A 75 41.429 113.954 97.499 1.00 1.00 C

ATOM 1250 N VAL A 76 43.275 111.842 95.232 1.00 1.00 N

ATOM 1251 CA VAL A 76 43.678 110.446 94.933 1.00 1.00 C

ATOM 1252 C VAL A 76 44.604 109.904 96.056 1.00 1.00 C

ATOM 1253 O VAL A 76 45.450 110.588 96.646 1.00 1.00 O

ATOM 1254 CB VAL A 76 44.293 110.248 93.505 1.00 1.00 C

ATOM 1255 CG1 VAL A 76 43.292 110.558 92.371 1.00 1.00 C

ATOM 1256 CG2 VAL A 76 45.638 110.962 93.244 1.00 1.00 C

ATOM 1257 N ASP A 77 44.451 108.608 96.266 1.00 1.00 N

ATOM 1258 CA ASP A 77 45.402 107.773 97.021 1.00 1.00 C

ATOM 1259 C ASP A 77 46.743 107.452 96.251 1.00 1.00 C

ATOM 1260 O ASP A 77 47.062 108.052 95.217 1.00 1.00 O

ATOM 1261 CB ASP A 77 44.494 106.563 97.381 1.00 1.00 C

ATOM 1262 CG ASP A 77 43.967 105.656 96.261 1.00 1.00 C

ATOM 1263 OD1 ASP A 77 44.383 105.804 95.093 1.00 1.00 O

ATOM 1264 OD2 ASP A 77 43.215 104.706 96.571 1.00 1.00 O

ATOM 1265 N ALA A 78 47.521 106.460 96.725 1.00 1.00 N

ATOM 1266 CA ALA A 78 48.647 105.859 95.945 1.00 1.00 C

ATOM 1267 C ALA A 78 48.371 105.283 94.504 1.00 1.00 C

ATOM 1268 O ALA A 78 49.331 104.934 93.807 1.00 1.00 O

ATOM 1269 CB ALA A 78 49.253 104.763 96.853 1.00 1.00 C

ATOM 1270 N ILE A 79 47.103 105.144 94.062 1.00 1.00 N

ATOM 1271 CA ILE A 79 46.737 104.381 92.818 1.00 1.00 C

ATOM 1272 C ILE A 79 45.897 105.181 91.785 1.00 1.00 C

ATOM 1273 O ILE A 79 45.411 104.571 90.835 1.00 1.00 O

ATOM 1274 CB ILE A 79 46.091 102.976 93.134 1.00 1.00 C

ATOM 1275 CG1 ILE A 79 44.569 102.929 93.481 1.00 1.00 C

ATOM 1276 CG2 ILE A 79 46.988 102.246 94.144 1.00 1.00 C

ATOM 1277 CD1 ILE A 79 43.940 101.552 93.748 1.00 1.00 C

ATOM 1278 N GLY A 80 45.667 106.491 91.952 1.00 1.00 N

ATOM 1279 CA GLY A 80 44.576 107.184 91.221 1.00 1.00 C

ATOM 1280 C GLY A 80 43.103 106.819 91.557 1.00 1.00 C

ATOM 1281 O GLY A 80 42.220 107.224 90.799 1.00 1.00 O

ATOM 1282 N PHE A 81 42.812 106.116 92.673 1.00 1.00 N

ATOM 1283 CA PHE A 81 41.426 105.925 93.165 1.00 1.00 C

ATOM 1284 C PHE A 81 40.982 107.228 93.888 1.00 1.00 C

ATOM 1285 O PHE A 81 41.427 107.559 94.992 1.00 1.00 O

ATOM 1286 CB PHE A 81 41.318 104.672 94.094 1.00 1.00 C

ATOM 1287 CG PHE A 81 40.644 103.400 93.567 1.00 1.00 C

ATOM 1288 CD1 PHE A 81 40.951 102.893 92.299 1.00 1.00 C

ATOM 1289 CD2 PHE A 81 39.787 102.675 94.405 1.00 1.00 C

ATOM 1290 CE1 PHE A 81 40.417 101.681 91.883 1.00 1.00 C

ATOM 1291 CE2 PHE A 81 39.246 101.466 93.977 1.00 1.00 C

ATOM 1292 CZ PHE A 81 39.579 100.959 92.726 1.00 1.00 C

ATOM 1293 N THR A 82 40.065 107.953 93.244 1.00 1.00 N

ATOM 1294 CA THR A 82 39.247 108.992 93.920 1.00 1.00 C

ATOM 1295 C THR A 82 38.229 108.335 94.932 1.00 1.00 C

ATOM 1296 O THR A 82 37.962 107.124 94.840 1.00 1.00 O

ATOM 1297 CB THR A 82 38.505 109.864 92.847 1.00 1.00 C

ATOM 1298 OG1 THR A 82 37.449 109.145 92.217 1.00 1.00 O

ATOM 1299 CG2 THR A 82 39.365 110.484 91.730 1.00 1.00 C

ATOM 1300 N PRO A 83 37.548 109.074 95.853 1.00 1.00 N

ATOM 1301 CA PRO A 83 36.311 108.571 96.513 1.00 1.00 C

ATOM 1302 C PRO A 83 35.206 107.921 95.606 1.00 1.00 C

ATOM 1303 O PRO A 83 34.582 106.946 96.023 1.00 1.00 O

ATOM 1304 CB PRO A 83 35.841 109.813 97.290 1.00 1.00 C

ATOM 1305 CG PRO A 83 37.106 110.642 97.525 1.00 1.00 C

ATOM 1306 CD PRO A 83 37.919 110.444 96.252 1.00 1.00 C

ATOM 1307 N LEU A 84 34.991 108.389 94.357 1.00 1.00 N

ATOM 1308 CA LEU A 84 34.078 107.707 93.391 1.00 1.00 C

ATOM 1309 C LEU A 84 34.510 106.272 92.951 1.00 1.00 C

ATOM 1310 O LEU A 84 33.673 105.366 92.966 1.00 1.00 O

ATOM 1311 CB LEU A 84 33.818 108.645 92.184 1.00 1.00 C

ATOM 1312 CG LEU A 84 32.636 108.234 91.264 1.00 1.00 C

ATOM 1313 CD1 LEU A 84 31.275 108.458 91.946 1.00 1.00 C

ATOM 1314 CD2 LEU A 84 32.699 108.990 89.928 1.00 1.00 C

ATOM 1315 N HIS A 85 35.793 106.047 92.611 1.00 1.00 N

ATOM 1316 CA HIS A 85 36.367 104.675 92.492 1.00 1.00 C

ATOM 1317 C HIS A 85 36.200 103.775 93.760 1.00 1.00 C

ATOM 1318 O HIS A 85 35.777 102.628 93.631 1.00 1.00 O

ATOM 1319 CB HIS A 85 37.870 104.755 92.149 1.00 1.00 C

ATOM 1320 CG HIS A 85 38.280 105.388 90.833 1.00 1.00 C

ATOM 1321 ND1 HIS A 85 38.619 106.726 90.705 1.00 1.00 N

ATOM 1322 CD2 HIS A 85 38.623 104.670 89.685 1.00 1.00 C

ATOM 1323 CE1 HIS A 85 39.156 106.688 89.449 1.00 1.00 C

ATOM 1324 NE2 HIS A 85 39.179 105.513 88.761 1.00 1.00 N

ATOM 1325 N LEU A 86 36.504 104.292 94.965 1.00 1.00 N

ATOM 1326 CA LEU A 86 36.250 103.576 96.243 1.00 1.00 C

ATOM 1327 C LEU A 86 34.745 103.268 96.561 1.00 1.00 C

ATOM 1328 O LEU A 86 34.433 102.132 96.922 1.00 1.00 O

ATOM 1329 CB LEU A 86 36.976 104.384 97.348 1.00 1.00 C

ATOM 1330 CG LEU A 86 37.190 103.654 98.696 1.00 1.00 C

ATOM 1331 CD1 LEU A 86 38.184 102.485 98.581 1.00 1.00 C

ATOM 1332 CD2 LEU A 86 37.731 104.666 99.710 1.00 1.00 C

ATOM 1333 N ALA A 87 33.818 104.228 96.371 1.00 1.00 N

ATOM 1334 CA ALA A 87 32.352 103.968 96.351 1.00 1.00 C

ATOM 1335 C ALA A 87 31.855 102.914 95.307 1.00 1.00 C

ATOM 1336 O ALA A 87 31.173 101.961 95.695 1.00 1.00 O

ATOM 1337 CB ALA A 87 31.673 105.335 96.161 1.00 1.00 C

ATOM 1338 N ALA A 88 32.248 103.045 94.020 1.00 1.00 N

ATOM 1339 CA ALA A 88 32.142 101.960 93.004 1.00 1.00 C

ATOM 1340 C ALA A 88 32.688 100.545 93.371 1.00 1.00 C

ATOM 1341 O ALA A 88 32.050 99.539 93.066 1.00 1.00 O

ATOM 1342 CB ALA A 88 32.829 102.506 91.740 1.00 1.00 C

ATOM 1343 N PHE A 89 33.846 100.498 94.038 1.00 1.00 N

ATOM 1344 CA PHE A 89 34.447 99.269 94.600 1.00 1.00 C

ATOM 1345 C PHE A 89 33.648 98.607 95.766 1.00 1.00 C

ATOM 1346 O PHE A 89 33.297 97.430 95.668 1.00 1.00 O

ATOM 1347 CB PHE A 89 35.865 99.738 95.015 1.00 1.00 C

ATOM 1348 CG PHE A 89 36.815 98.624 95.397 1.00 1.00 C

ATOM 1349 CD1 PHE A 89 37.660 98.106 94.412 1.00 1.00 C

ATOM 1350 CD2 PHE A 89 36.883 98.143 96.696 1.00 1.00 C

ATOM 1351 CE1 PHE A 89 38.592 97.144 94.753 1.00 1.00 C

ATOM 1352 CE2 PHE A 89 37.818 97.168 97.027 1.00 1.00 C

ATOM 1353 CZ PHE A 89 38.719 96.722 96.058 1.00 1.00 C

ATOM 1354 N ILE A 90 33.376 99.341 96.864 1.00 1.00 N

ATOM 1355 CA ILE A 90 32.535 98.828 97.993 1.00 1.00 C

ATOM 1356 C ILE A 90 31.021 98.559 97.646 1.00 1.00 C

ATOM 1357 O ILE A 90 30.350 97.821 98.369 1.00 1.00 O

ATOM 1358 CB ILE A 90 32.724 99.684 99.301 1.00 1.00 C

ATOM 1359 CG1 ILE A 90 32.132 101.124 99.243 1.00 1.00 C

ATOM 1360 CG2 ILE A 90 34.195 99.706 99.800 1.00 1.00 C

ATOM 1361 CD1 ILE A 90 31.953 101.827 100.600 1.00 1.00 C

ATOM 1362 N GLY A 91 30.468 99.157 96.576 1.00 1.00 N

ATOM 1363 CA GLY A 91 29.016 99.107 96.279 1.00 1.00 C

ATOM 1364 C GLY A 91 28.089 100.015 97.113 1.00 1.00 C

ATOM 1365 O GLY A 91 26.936 99.636 97.337 1.00 1.00 O

ATOM 1366 N HIS A 92 28.550 101.210 97.530 1.00 1.00 N

ATOM 1367 CA HIS A 92 27.699 102.179 98.263 1.00 1.00 C

ATOM 1368 C HIS A 92 27.059 103.167 97.244 1.00 1.00 C

ATOM 1369 O HIS A 92 27.675 104.140 96.791 1.00 1.00 O

ATOM 1370 CB HIS A 92 28.492 102.870 99.402 1.00 1.00 C

ATOM 1371 CG HIS A 92 27.595 103.459 100.500 1.00 1.00 C

ATOM 1372 ND1 HIS A 92 26.589 104.395 100.285 1.00 1.00 N

ATOM 1373 CD2 HIS A 92 27.635 103.088 101.854 1.00 1.00 C

ATOM 1374 CE1 HIS A 92 26.113 104.513 101.566 1.00 1.00 C

ATOM 1375 NE2 HIS A 92 26.680 103.783 102.573 1.00 1.00 N

ATOM 1376 N LEU A 93 25.796 102.874 96.903 1.00 1.00 N

ATOM 1377 CA LEU A 93 25.000 103.658 95.924 1.00 1.00 C

ATOM 1378 C LEU A 93 24.791 105.162 96.302 1.00 1.00 C

ATOM 1379 O LEU A 93 25.174 106.031 95.518 1.00 1.00 O

ATOM 1380 CB LEU A 93 23.683 102.852 95.719 1.00 1.00 C

ATOM 1381 CG LEU A 93 22.847 103.199 94.463 1.00 1.00 C

ATOM 1382 CD1 LEU A 93 23.528 102.698 93.178 1.00 1.00 C

ATOM 1383 CD2 LEU A 93 21.452 102.559 94.569 1.00 1.00 C

ATOM 1384 N GLU A 94 24.271 105.463 97.508 1.00 1.00 N

ATOM 1385 CA GLU A 94 24.096 106.860 98.007 1.00 1.00 C

ATOM 1386 C GLU A 94 25.401 107.724 98.129 1.00 1.00 C

ATOM 1387 O GLU A 94 25.380 108.903 97.760 1.00 1.00 O

ATOM 1388 CB GLU A 94 23.321 106.824 99.352 1.00 1.00 C

ATOM 1389 CG GLU A 94 21.858 106.313 99.287 1.00 1.00 C

ATOM 1390 CD GLU A 94 21.093 106.471 100.591 1.00 1.00 C

ATOM 1391 OE1 GLU A 94 21.082 105.518 101.398 1.00 1.00 O

ATOM 1392 OE2 GLU A 94 20.495 107.545 100.809 1.00 1.00 O

ATOM 1393 N ILE A 95 26.539 107.160 98.597 1.00 1.00 N

ATOM 1394 CA ILE A 95 27.880 107.831 98.526 1.00 1.00 C

ATOM 1395 C ILE A 95 28.316 108.158 97.058 1.00 1.00 C

ATOM 1396 O ILE A 95 28.626 109.321 96.784 1.00 1.00 O

ATOM 1397 CB ILE A 95 28.950 107.087 99.405 1.00 1.00 C

ATOM 1398 CG1 ILE A 95 28.666 107.287 100.922 1.00 1.00 C

ATOM 1399 CG2 ILE A 95 30.415 107.500 99.113 1.00 1.00 C

ATOM 1400 CD1 ILE A 95 29.494 106.416 101.875 1.00 1.00 C

ATOM 1401 N ALA A 96 28.302 107.189 96.120 1.00 1.00 N

ATOM 1402 CA ALA A 96 28.452 107.483 94.669 1.00 1.00 C

ATOM 1403 C ALA A 96 27.483 108.568 94.077 1.00 1.00 C

ATOM 1404 O ALA A 96 27.967 109.511 93.447 1.00 1.00 O

ATOM 1405 CB ALA A 96 28.394 106.132 93.934 1.00 1.00 C

ATOM 1406 N GLU A 97 26.163 108.500 94.351 1.00 1.00 N

ATOM 1407 CA GLU A 97 25.186 109.584 94.026 1.00 1.00 C

ATOM 1408 C GLU A 97 25.536 111.006 94.588 1.00 1.00 C

ATOM 1409 O GLU A 97 25.600 111.954 93.799 1.00 1.00 O

ATOM 1410 CB GLU A 97 23.753 109.154 94.453 1.00 1.00 C

ATOM 1411 CG GLU A 97 23.138 107.950 93.692 1.00 1.00 C

ATOM 1412 CD GLU A 97 21.747 107.509 94.125 1.00 1.00 C

ATOM 1413 OE1 GLU A 97 21.431 107.541 95.333 1.00 1.00 O

ATOM 1414 OE2 GLU A 97 20.968 107.102 93.239 1.00 1.00 O

ATOM 1415 N VAL A 98 25.790 111.183 95.906 1.00 1.00 N

ATOM 1416 CA VAL A 98 26.264 112.495 96.461 1.00 1.00 C

ATOM 1417 C VAL A 98 27.667 112.982 95.948 1.00 1.00 C

ATOM 1418 O VAL A 98 27.803 114.162 95.617 1.00 1.00 O

ATOM 1419 CB VAL A 98 26.057 112.596 98.011 1.00 1.00 C

ATOM 1420 CG1 VAL A 98 27.067 111.793 98.854 1.00 1.00 C

ATOM 1421 CG2 VAL A 98 26.058 114.064 98.494 1.00 1.00 C

ATOM 1422 N LEU A 99 28.681 112.104 95.818 1.00 1.00 N

ATOM 1423 CA LEU A 99 29.941 112.411 95.074 1.00 1.00 C

ATOM 1424 C LEU A 99 29.735 112.957 93.618 1.00 1.00 C

ATOM 1425 O LEU A 99 30.256 114.027 93.294 1.00 1.00 O

ATOM 1426 CB LEU A 99 30.847 111.146 95.093 1.00 1.00 C

ATOM 1427 CG LEU A 99 31.485 110.752 96.452 1.00 1.00 C

ATOM 1428 CD1 LEU A 99 32.060 109.336 96.357 1.00 1.00 C

ATOM 1429 CD2 LEU A 99 32.603 111.721 96.864 1.00 1.00 C

ATOM 1430 N LEU A 100 28.939 112.272 92.775 1.00 1.00 N

ATOM 1431 CA LEU A 100 28.493 112.789 91.444 1.00 1.00 C

ATOM 1432 C LEU A 100 27.727 114.160 91.474 1.00 1.00 C

ATOM 1433 O LEU A 100 28.058 115.051 90.687 1.00 1.00 O

ATOM 1434 CB LEU A 100 27.655 111.677 90.747 1.00 1.00 C

ATOM 1435 CG LEU A 100 28.406 110.368 90.373 1.00 1.00 C

ATOM 1436 CD1 LEU A 100 27.426 109.194 90.204 1.00 1.00 C

ATOM 1437 CD2 LEU A 100 29.288 110.532 89.123 1.00 1.00 C

ATOM 1438 N LYS A 101 26.767 114.364 92.403 1.00 1.00 N

ATOM 1439 CA LYS A 101 26.173 115.711 92.688 1.00 1.00 C

ATOM 1440 C LYS A 101 27.179 116.859 93.054 1.00 1.00 C

ATOM 1441 O LYS A 101 26.987 117.988 92.598 1.00 1.00 O

ATOM 1442 CB LYS A 101 25.089 115.583 93.797 1.00 1.00 C

ATOM 1443 CG LYS A 101 23.820 114.789 93.407 1.00 1.00 C

ATOM 1444 CD LYS A 101 22.819 114.673 94.575 1.00 1.00 C

ATOM 1445 CE LYS A 101 21.640 113.746 94.230 1.00 1.00 C

ATOM 1446 NZ LYS A 101 20.670 113.719 95.358 1.00 1.00 N

ATOM 1447 N HIS A 102 28.241 116.584 93.834 1.00 1.00 N

ATOM 1448 CA HIS A 102 29.349 117.562 94.077 1.00 1.00 C

ATOM 1449 C HIS A 102 30.543 117.503 93.050 1.00 1.00 C

ATOM 1450 O HIS A 102 31.676 117.860 93.385 1.00 1.00 O

ATOM 1451 CB HIS A 102 29.800 117.375 95.555 1.00 1.00 C

ATOM 1452 CG HIS A 102 28.814 117.911 96.596 1.00 1.00 C

ATOM 1453 ND1 HIS A 102 28.906 119.170 97.169 1.00 1.00 N

ATOM 1454 CD2 HIS A 102 27.738 117.195 97.144 1.00 1.00 C

ATOM 1455 CE1 HIS A 102 27.850 119.091 98.040 1.00 1.00 C

ATOM 1456 NE2 HIS A 102 27.093 117.951 98.102 1.00 1.00 N

ATOM 1457 N GLY A 103 30.288 117.127 91.782 1.00 1.00 N

ATOM 1458 CA GLY A 103 31.310 117.150 90.702 1.00 1.00 C

ATOM 1459 C GLY A 103 32.435 116.094 90.690 1.00 1.00 C

ATOM 1460 O GLY A 103 33.506 116.395 90.157 1.00 1.00 O

ATOM 1461 N ALA A 104 32.217 114.866 91.197 1.00 1.00 N

ATOM 1462 CA ALA A 104 33.194 113.759 91.042 1.00 1.00 C

ATOM 1463 C ALA A 104 33.286 113.269 89.571 1.00 1.00 C

ATOM 1464 O ALA A 104 32.272 112.912 88.957 1.00 1.00 O

ATOM 1465 CB ALA A 104 32.794 112.606 91.973 1.00 1.00 C

ATOM 1466 N ASP A 105 34.505 113.273 89.010 1.00 1.00 N

ATOM 1467 CA ASP A 105 34.715 112.974 87.575 1.00 1.00 C

ATOM 1468 C ASP A 105 34.461 111.463 87.254 1.00 1.00 C

ATOM 1469 O ASP A 105 35.241 110.573 87.608 1.00 1.00 O

ATOM 1470 CB ASP A 105 36.116 113.508 87.181 1.00 1.00 C

ATOM 1471 CG ASP A 105 36.514 113.377 85.705 1.00 1.00 C

ATOM 1472 OD1 ASP A 105 35.664 113.030 84.852 1.00 1.00 O

ATOM 1473 OD2 ASP A 105 37.700 113.612 85.397 1.00 1.00 O

ATOM 1474 N VAL A 106 33.357 111.209 86.532 1.00 1.00 N

ATOM 1475 CA VAL A 106 33.051 109.873 85.932 1.00 1.00 C

ATOM 1476 C VAL A 106 34.139 109.307 84.940 1.00 1.00 C

ATOM 1477 O VAL A 106 34.338 108.092 84.878 1.00 1.00 O

ATOM 1478 CB VAL A 106 31.583 109.922 85.363 1.00 1.00 C

ATOM 1479 CG1 VAL A 106 31.443 110.479 83.926 1.00 1.00 C

ATOM 1480 CG2 VAL A 106 30.873 108.556 85.435 1.00 1.00 C

ATOM 1481 N ASN A 107 34.848 110.189 84.205 1.00 1.00 N

ATOM 1482 CA ASN A 107 36.022 109.817 83.369 1.00 1.00 C

ATOM 1483 C ASN A 107 37.404 109.730 84.108 1.00 1.00 C

ATOM 1484 O ASN A 107 38.380 109.351 83.453 1.00 1.00 O

ATOM 1485 CB ASN A 107 36.133 110.846 82.207 1.00 1.00 C

ATOM 1486 CG ASN A 107 34.945 110.931 81.238 1.00 1.00 C

ATOM 1487 OD1 ASN A 107 34.402 109.936 80.770 1.00 1.00 O

ATOM 1488 ND2 ASN A 107 34.517 112.121 80.897 1.00 1.00 N

ATOM 1489 N ALA A 108 37.528 110.037 85.422 1.00 1.00 N

ATOM 1490 CA ALA A 108 38.808 109.927 86.166 1.00 1.00 C

ATOM 1491 C ALA A 108 39.379 108.484 86.153 1.00 1.00 C

ATOM 1492 O ALA A 108 38.706 107.533 86.557 1.00 1.00 O

ATOM 1493 CB ALA A 108 38.595 110.403 87.615 1.00 1.00 C

ATOM 1494 N GLN A 109 40.604 108.334 85.644 1.00 1.00 N

ATOM 1495 CA GLN A 109 41.257 107.010 85.512 1.00 1.00 C

ATOM 1496 C GLN A 109 42.229 106.763 86.695 1.00 1.00 C

ATOM 1497 O GLN A 109 43.003 107.644 87.092 1.00 1.00 O

ATOM 1498 CB GLN A 109 42.002 106.933 84.155 1.00 1.00 C

ATOM 1499 CG GLN A 109 41.076 106.810 82.910 1.00 1.00 C

ATOM 1500 CD GLN A 109 41.696 106.804 81.503 1.00 1.00 C

ATOM 1501 OE1 GLN A 109 41.028 107.094 80.518 1.00 1.00 O

ATOM 1502 NE2 GLN A 109 42.954 106.476 81.315 1.00 1.00 N

ATOM 1503 N ASP A 110 42.248 105.521 87.195 1.00 1.00 N

ATOM 1504 CA ASP A 110 43.322 105.053 88.100 1.00 1.00 C

ATOM 1505 C ASP A 110 44.716 104.900 87.382 1.00 1.00 C

ATOM 1506 O ASP A 110 44.865 105.056 86.162 1.00 1.00 O

ATOM 1507 CB ASP A 110 42.782 103.811 88.873 1.00 1.00 C

ATOM 1508 CG ASP A 110 42.720 102.448 88.180 1.00 1.00 C

ATOM 1509 OD1 ASP A 110 43.266 102.268 87.068 1.00 1.00 O

ATOM 1510 OD2 ASP A 110 42.118 101.532 88.773 1.00 1.00 O

ATOM 1511 N LYS A 111 45.743 104.540 88.159 1.00 1.00 N

ATOM 1512 CA LYS A 111 47.072 104.085 87.657 1.00 1.00 C

ATOM 1513 C LYS A 111 47.102 103.052 86.474 1.00 1.00 C

ATOM 1514 O LYS A 111 48.054 103.074 85.689 1.00 1.00 O

ATOM 1515 CB LYS A 111 47.809 103.570 88.929 1.00 1.00 C

ATOM 1516 CG LYS A 111 49.270 103.109 88.741 1.00 1.00 C

ATOM 1517 CD LYS A 111 49.933 102.729 90.080 1.00 1.00 C

ATOM 1518 CE LYS A 111 51.341 102.149 89.870 1.00 1.00 C

ATOM 1519 NZ LYS A 111 51.985 101.934 91.192 1.00 1.00 N

ATOM 1520 N PHE A 112 46.100 102.163 86.351 1.00 1.00 N

ATOM 1521 CA PHE A 112 46.005 101.179 85.227 1.00 1.00 C

ATOM 1522 C PHE A 112 44.873 101.507 84.187 1.00 1.00 C

ATOM 1523 O PHE A 112 44.355 100.605 83.516 1.00 1.00 O

ATOM 1524 CB PHE A 112 45.881 99.757 85.862 1.00 1.00 C

ATOM 1525 CG PHE A 112 47.108 99.333 86.692 1.00 1.00 C

ATOM 1526 CD1 PHE A 112 48.252 98.820 86.072 1.00 1.00 C

ATOM 1527 CD2 PHE A 112 47.154 99.668 88.048 1.00 1.00 C

ATOM 1528 CE1 PHE A 112 49.428 98.655 86.800 1.00 1.00 C

ATOM 1529 CE2 PHE A 112 48.336 99.536 88.760 1.00 1.00 C

ATOM 1530 CZ PHE A 112 49.469 99.013 88.144 1.00 1.00 C

ATOM 1531 N GLY A 113 44.527 102.798 84.008 1.00 1.00 N

ATOM 1532 CA GLY A 113 43.459 103.235 83.077 1.00 1.00 C

ATOM 1533 C GLY A 113 41.976 102.974 83.419 1.00 1.00 C

ATOM 1534 O GLY A 113 41.157 103.108 82.508 1.00 1.00 O

ATOM 1535 N LYS A 114 41.603 102.610 84.659 1.00 1.00 N

ATOM 1536 CA LYS A 114 40.211 102.184 84.975 1.00 1.00 C

ATOM 1537 C LYS A 114 39.384 103.370 85.527 1.00 1.00 C

ATOM 1538 O LYS A 114 39.811 104.045 86.467 1.00 1.00 O

ATOM 1539 CB LYS A 114 40.195 100.984 85.969 1.00 1.00 C

ATOM 1540 CG LYS A 114 41.027 99.722 85.626 1.00 1.00 C

ATOM 1541 CD LYS A 114 40.854 99.243 84.170 1.00 1.00 C

ATOM 1542 CE LYS A 114 41.656 97.976 83.844 1.00 1.00 C

ATOM 1543 NZ LYS A 114 41.727 97.845 82.365 1.00 1.00 N

ATOM 1544 N THR A 115 38.186 103.599 84.975 1.00 1.00 N

ATOM 1545 CA THR A 115 37.194 104.538 85.578 1.00 1.00 C

ATOM 1546 C THR A 115 36.445 103.888 86.787 1.00 1.00 C

ATOM 1547 O THR A 115 36.443 102.665 86.972 1.00 1.00 O

ATOM 1548 CB THR A 115 36.194 105.080 84.504 1.00 1.00 C

ATOM 1549 OG1 THR A 115 35.328 104.054 84.030 1.00 1.00 O

ATOM 1550 CG2 THR A 115 36.827 105.758 83.278 1.00 1.00 C

ATOM 1551 N ALA A 116 35.714 104.694 87.580 1.00 1.00 N

ATOM 1552 CA ALA A 116 34.685 104.160 88.517 1.00 1.00 C

ATOM 1553 C ALA A 116 33.630 103.164 87.906 1.00 1.00 C

ATOM 1554 O ALA A 116 33.269 102.181 88.559 1.00 1.00 O

ATOM 1555 CB ALA A 116 34.040 105.377 89.199 1.00 1.00 C

ATOM 1556 N PHE A 117 33.179 103.367 86.646 1.00 1.00 N

ATOM 1557 CA PHE A 117 32.404 102.342 85.888 1.00 1.00 C

ATOM 1558 C PHE A 117 33.148 100.969 85.715 1.00 1.00 C

ATOM 1559 O PHE A 117 32.610 99.947 86.151 1.00 1.00 O

ATOM 1560 CB PHE A 117 31.901 102.976 84.557 1.00 1.00 C

ATOM 1561 CG PHE A 117 31.067 102.016 83.688 1.00 1.00 C

ATOM 1562 CD1 PHE A 117 29.747 101.710 84.034 1.00 1.00 C

ATOM 1563 CD2 PHE A 117 31.688 101.307 82.653 1.00 1.00 C

ATOM 1564 CE1 PHE A 117 29.068 100.691 83.370 1.00 1.00 C

ATOM 1565 CE2 PHE A 117 31.002 100.297 81.984 1.00 1.00 C

ATOM 1566 CZ PHE A 117 29.693 99.990 82.343 1.00 1.00 C

ATOM 1567 N ASP A 118 34.369 100.957 85.139 1.00 1.00 N

ATOM 1568 CA ASP A 118 35.256 99.750 85.098 1.00 1.00 C

ATOM 1569 C ASP A 118 35.454 99.009 86.467 1.00 1.00 C

ATOM 1570 O ASP A 118 35.343 97.781 86.539 1.00 1.00 O

ATOM 1571 CB ASP A 118 36.647 100.149 84.532 1.00 1.00 C

ATOM 1572 CG ASP A 118 36.719 100.745 83.130 1.00 1.00 C

ATOM 1573 OD1 ASP A 118 36.106 100.189 82.195 1.00 1.00 O

ATOM 1574 OD2 ASP A 118 37.416 101.769 82.962 1.00 1.00 O

ATOM 1575 N ILE A 119 35.693 99.773 87.549 1.00 1.00 N

ATOM 1576 CA ILE A 119 35.734 99.253 88.942 1.00 1.00 C

ATOM 1577 C ILE A 119 34.380 98.625 89.408 1.00 1.00 C

ATOM 1578 O ILE A 119 34.396 97.446 89.758 1.00 1.00 O

ATOM 1579 CB ILE A 119 36.338 100.348 89.888 1.00 1.00 C

ATOM 1580 CG1 ILE A 119 37.773 100.831 89.510 1.00 1.00 C

ATOM 1581 CG2 ILE A 119 36.360 99.919 91.367 1.00 1.00 C

ATOM 1582 CD1 ILE A 119 38.837 99.743 89.260 1.00 1.00 C

ATOM 1583 N SER A 120 33.235 99.336 89.384 1.00 1.00 N

ATOM 1584 CA SER A 120 31.894 98.715 89.639 1.00 1.00 C

ATOM 1585 C SER A 120 31.525 97.435 88.807 1.00 1.00 C

ATOM 1586 O SER A 120 31.030 96.470 89.391 1.00 1.00 O

ATOM 1587 CB SER A 120 30.795 99.802 89.569 1.00 1.00 C

ATOM 1588 OG SER A 120 30.701 100.404 88.278 1.00 1.00 O

ATOM 1589 N ILE A 121 31.833 97.379 87.495 1.00 1.00 N

ATOM 1590 CA ILE A 121 31.741 96.128 86.669 1.00 1.00 C

ATOM 1591 C ILE A 121 32.711 94.986 87.147 1.00 1.00 C

ATOM 1592 O ILE A 121 32.230 93.900 87.485 1.00 1.00 O

ATOM 1593 CB ILE A 121 31.862 96.495 85.143 1.00 1.00 C

ATOM 1594 CG1 ILE A 121 30.690 97.365 84.598 1.00 1.00 C

ATOM 1595 CG2 ILE A 121 32.075 95.288 84.188 1.00 1.00 C

ATOM 1596 CD1 ILE A 121 29.285 96.726 84.595 1.00 1.00 C

ATOM 1597 N GLY A 122 34.044 95.206 87.206 1.00 1.00 N

ATOM 1598 CA GLY A 122 34.991 94.236 87.850 1.00 1.00 C

ATOM 1599 C GLY A 122 34.702 93.751 89.300 1.00 1.00 C

ATOM 1600 O GLY A 122 34.992 92.608 89.660 1.00 1.00 O

ATOM 1601 N ASN A 123 34.100 94.630 90.104 1.00 1.00 N

ATOM 1602 CA ASN A 123 33.602 94.329 91.472 1.00 1.00 C

ATOM 1603 C ASN A 123 32.072 93.951 91.543 1.00 1.00 C

ATOM 1604 O ASN A 123 31.468 94.085 92.609 1.00 1.00 O

ATOM 1605 CB ASN A 123 33.996 95.585 92.314 1.00 1.00 C

ATOM 1606 CG ASN A 123 35.507 95.705 92.612 1.00 1.00 C

ATOM 1607 OD1 ASN A 123 36.063 95.137 93.545 1.00 1.00 O

ATOM 1608 ND2 ASN A 123 36.242 96.385 91.771 1.00 1.00 N

ATOM 1609 N GLY A 124 31.441 93.417 90.469 1.00 1.00 N

ATOM 1610 CA GLY A 124 30.008 92.977 90.467 1.00 1.00 C

ATOM 1611 C GLY A 124 28.891 93.887 91.041 1.00 1.00 C

ATOM 1612 O GLY A 124 27.872 93.385 91.519 1.00 1.00 O

ATOM 1613 N ASN A 125 29.073 95.213 91.014 1.00 1.00 N

ATOM 1614 CA ASN A 125 28.148 96.175 91.666 1.00 1.00 C

ATOM 1615 C ASN A 125 27.203 96.777 90.590 1.00 1.00 C

ATOM 1616 O ASN A 125 27.371 97.914 90.137 1.00 1.00 O

ATOM 1617 CB ASN A 125 28.985 97.217 92.465 1.00 1.00 C

ATOM 1618 CG ASN A 125 29.845 96.701 93.629 1.00 1.00 C

ATOM 1619 OD1 ASN A 125 31.047 96.912 93.689 1.00 1.00 O

ATOM 1620 ND2 ASN A 125 29.287 96.017 94.597 1.00 1.00 N

ATOM 1621 N GLU A 126 26.201 95.972 90.194 1.00 1.00 N

ATOM 1622 CA GLU A 126 25.384 96.224 88.973 1.00 1.00 C

ATOM 1623 C GLU A 126 24.501 97.514 89.031 1.00 1.00 C

ATOM 1624 O GLU A 126 24.610 98.361 88.141 1.00 1.00 O

ATOM 1625 CB GLU A 126 24.571 94.945 88.610 1.00 1.00 C

ATOM 1626 CG GLU A 126 25.318 93.578 88.517 1.00 1.00 C

ATOM 1627 CD GLU A 126 26.575 93.493 87.654 1.00 1.00 C

ATOM 1628 OE1 GLU A 126 26.623 94.095 86.561 1.00 1.00 O

ATOM 1629 OE2 GLU A 126 27.520 92.791 88.071 1.00 1.00 O

ATOM 1630 N ASP A 127 23.727 97.719 90.116 1.00 1.00 N

ATOM 1631 CA ASP A 127 23.061 99.019 90.440 1.00 1.00 C

ATOM 1632 C ASP A 127 23.987 100.293 90.413 1.00 1.00 C

ATOM 1633 O ASP A 127 23.599 101.340 89.886 1.00 1.00 O

ATOM 1634 CB ASP A 127 22.390 98.892 91.842 1.00 1.00 C

ATOM 1635 CG ASP A 127 21.256 97.882 92.062 1.00 1.00 C

ATOM 1636 OD1 ASP A 127 20.854 97.160 91.127 1.00 1.00 O

ATOM 1637 OD2 ASP A 127 20.773 97.806 93.212 1.00 1.00 O

ATOM 1638 N LEU A 128 25.213 100.202 90.968 1.00 1.00 N

ATOM 1639 CA LEU A 128 26.226 101.293 90.891 1.00 1.00 C

ATOM 1640 C LEU A 128 26.847 101.512 89.466 1.00 1.00 C

ATOM 1641 O LEU A 128 26.920 102.655 89.003 1.00 1.00 O

ATOM 1642 CB LEU A 128 27.261 101.085 92.035 1.00 1.00 C

ATOM 1643 CG LEU A 128 27.830 102.386 92.660 1.00 1.00 C

ATOM 1644 CD1 LEU A 128 28.487 102.078 94.010 1.00 1.00 C

ATOM 1645 CD2 LEU A 128 28.828 103.100 91.740 1.00 1.00 C

ATOM 1646 N ALA A 129 27.218 100.442 88.735 1.00 1.00 N

ATOM 1647 CA ALA A 129 27.453 100.504 87.267 1.00 1.00 C

ATOM 1648 C ALA A 129 26.311 101.132 86.392 1.00 1.00 C

ATOM 1649 O ALA A 129 26.615 101.952 85.522 1.00 1.00 O

ATOM 1650 CB ALA A 129 27.810 99.076 86.819 1.00 1.00 C

ATOM 1651 N GLU A 130 25.023 100.824 86.653 1.00 1.00 N

ATOM 1652 CA GLU A 130 23.859 101.547 86.058 1.00 1.00 C

ATOM 1653 C GLU A 130 23.811 103.093 86.319 1.00 1.00 C

ATOM 1654 O GLU A 130 23.653 103.847 85.353 1.00 1.00 O

ATOM 1655 CB GLU A 130 22.532 100.866 86.494 1.00 1.00 C

ATOM 1656 CG GLU A 130 22.251 99.462 85.893 1.00 1.00 C

ATOM 1657 CD GLU A 130 20.907 98.823 86.231 1.00 1.00 C

ATOM 1658 OE1 GLU A 130 19.998 99.494 86.769 1.00 1.00 O

ATOM 1659 OE2 GLU A 130 20.751 97.626 85.914 1.00 1.00 O

ATOM 1660 N ILE A 131 23.981 103.589 87.569 1.00 1.00 N

ATOM 1661 CA ILE A 131 24.113 105.068 87.841 1.00 1.00 C

ATOM 1662 C ILE A 131 25.313 105.766 87.100 1.00 1.00 C

ATOM 1663 O ILE A 131 25.151 106.860 86.554 1.00 1.00 O

ATOM 1664 CB ILE A 131 24.008 105.490 89.359 1.00 1.00 C

ATOM 1665 CG1 ILE A 131 25.303 105.318 90.211 1.00 1.00 C

ATOM 1666 CG2 ILE A 131 22.788 104.863 90.080 1.00 1.00 C

ATOM 1667 CD1 ILE A 131 25.333 106.078 91.544 1.00 1.00 C

ATOM 1668 N LEU A 132 26.493 105.119 87.078 1.00 1.00 N

ATOM 1669 CA LEU A 132 27.700 105.607 86.356 1.00 1.00 C

ATOM 1670 C LEU A 132 27.606 105.539 84.797 1.00 1.00 C

ATOM 1671 O LEU A 132 27.971 106.515 84.144 1.00 1.00 O

ATOM 1672 CB LEU A 132 28.932 104.831 86.897 1.00 1.00 C

ATOM 1673 CG LEU A 132 29.300 105.055 88.389 1.00 1.00 C

ATOM 1674 CD1 LEU A 132 30.301 103.992 88.839 1.00 1.00 C

ATOM 1675 CD2 LEU A 132 29.908 106.440 88.651 1.00 1.00 C

ATOM 1676 N GLN A 133 27.082 104.450 84.196 1.00 1.00 N

ATOM 1677 CA GLN A 133 26.665 104.431 82.760 1.00 1.00 C

ATOM 1678 C GLN A 133 25.609 105.500 82.314 1.00 1.00 C

ATOM 1679 O GLN A 133 25.671 105.985 81.182 1.00 1.00 O

ATOM 1680 CB GLN A 133 26.179 102.987 82.446 1.00 1.00 C

ATOM 1681 CG GLN A 133 25.949 102.631 80.949 1.00 1.00 C

ATOM 1682 CD GLN A 133 27.106 102.839 79.959 1.00 1.00 C

ATOM 1683 OE1 GLN A 133 26.954 103.457 78.911 1.00 1.00 O

ATOM 1684 NE2 GLN A 133 28.286 102.331 80.221 1.00 1.00 N

ATOM 1685 N LYS A 134 24.662 105.878 83.190 1.00 1.00 N

ATOM 1686 CA LYS A 134 23.768 107.050 82.971 1.00 1.00 C

ATOM 1687 C LYS A 134 24.420 108.480 82.852 1.00 1.00 C

ATOM 1688 O LYS A 134 23.737 109.391 82.372 1.00 1.00 O

ATOM 1689 CB LYS A 134 22.680 107.018 84.093 1.00 1.00 C

ATOM 1690 CG LYS A 134 21.236 107.081 83.552 1.00 1.00 C

ATOM 1691 CD LYS A 134 20.192 107.220 84.677 1.00 1.00 C

ATOM 1692 CE LYS A 134 18.754 107.135 84.135 1.00 1.00 C

ATOM 1693 NZ LYS A 134 17.796 107.565 85.188 1.00 1.00 N

ATOM 1694 N LEU A 135 25.669 108.692 83.309 1.00 1.00 N

ATOM 1695 CA LEU A 135 26.351 110.017 83.289 1.00 1.00 C

ATOM 1696 C LEU A 135 27.689 110.005 82.498 1.00 1.00 C

ATOM 1697 O LEU A 135 28.108 111.101 82.057 1.00 1.00 O

ATOM 1698 CB LEU A 135 26.595 110.483 84.752 1.00 1.00 C

ATOM 1699 CG LEU A 135 25.344 110.839 85.602 1.00 1.00 C

ATOM 1700 CD1 LEU A 135 25.749 111.050 87.066 1.00 1.00 C

ATOM 1701 CD2 LEU A 135 24.611 112.090 85.088 1.00 1.00 C

ATOM 1702 OXT LEU A 135 28.341 108.947 82.328 1.00 0.00 O

END
